# Characterisation of the virome of Culicoides brevitarsis Kieffer (Diptera: Ceratopogonidae), a vector of bluetongue virus in Australia

**DOI:** 10.1099/jgv.0.002076

**Published:** 2025-02-20

**Authors:** Stephen R. Sharpe, Mukund Madhav, Melissa J. Klein, Kim R. Blasdell, Prasad N. Paradkar, Stacey E. Lynch, Debbie Eagles, Adam J. López-Denman, Khandaker Asif Ahmed

**Affiliations:** 1 CSIRO Australian Centre for Disease Preparedness (ACDP), East Geelong, VIC 3220, Australia

**Keywords:** arbovirus, *Culicoides*, insect-specific virus, RNA virus, vector

## Abstract

*Culicoides* spp., a common biting midge genus, are haematophagous insects that can transmit pathogens to humans and other animals. Some species transmit arboviruses, including bluetongue virus, epizootic haemorrhagic disease virus, African horse sickness virus and Schmallenberg virus to vertebrates, which can be detrimental to livestock and wild animals. *Culicoides* spp. can also have a diversity of insect-specific viruses (ISVs) that can only be transmitted between insects and others related to known arboviruses. For *Culicoides brevitarsis* and other *Culicoides* spp. in Australia, the virome is largely unexplored. We used high-throughput sequencing to characterise the virome of *C. brevitarsis* collected from Casino, New South Wales, Australia. For virus detection, the total RNA was extracted from pools of *C. brevitarsis* followed by rRNA depletion and Illumina short-read-based RNA sequencing. The reads were quality-checked, filtered and assembled into contigs, compared with the non-redundant protein and conserved domain databases for viral detection and genome organisation, respectively. The phylogenetic analysis was used to further characterise the viruses. We detected new virus diversity including ten viruses belonging to eight different families with complete or near-complete coding regions. Seven of these were novel virus species belonging to the families: *Chuviridae*, *Orthomyxoviridae*, *Peribunyaviridae*, *Qinviridae*, *Rhabdoviridae* and *Solemoviridae*. In addition, the novel *Peribunyaviridae* virus should also be considered part of a new genus. Whilst most of the detected viruses grouped into families with viruses that can infect insects, animals or both, the novel species of *Solemoviridae* was closely related to an economically important plant pathogen, the sugarcane yellow leaf virus. Our quantitative PCR-based screening confirmed the absence of any *Wolbachia* endosymbiont within the collected samples. Furthermore, we detected fragments of three more virus families known to infect fungi and plants. The detection of potential arboviruses and ISVs in *Culicoides* spp. is important in understanding virus epidemiology.

## Graphical abstract

A metatranscriptomics study of a dominant bluetongue virus vector, *Culicoides brevitarsis* from Australia, identified 10 complete/near-complete viral genomes including 7 novel species.

## Introduction

*Culicoides* are the largest genus in the Ceratopogonidae family. These haematophagous insects are found worldwide with >1300 described species and many more undescribed species [1]. *Culicoides* spp. are of significant public health and veterinary concern due to their irritating bites, causing allergic reactions, and their role as vectors in the transmission of microbes to humans, birds and livestock. Most of the known viruses transmitted by *Culicoides* are members of the *Sedoreoviridae* [bluetongue virus (BTV), epizootic haemorrhagic disease virus (EHDV) and African horse sickness virus (AHSV)], *Rhabdoviridae* (bovine ephemeral fever virus), and *Peribunyaviridae* family [Schmallenberg virus (SBV), Oropouche virus (OROV) and Akabane virus (AKAV)] [2]. The geographical distribution of these viruses correlates with the distribution of their respective vector species [3].

Apart from arboviruses, *Culicoides* spp. virome may contain insect-specific viruses (ISVs) those that infect insects and not vertebrates. Some ISVs have been found to influence arbovirus replication and the vector competence of mosquitoes. For instance, studies with *Culex* mosquitoes have shown that infection with the ISV *Culex* flavivirus reduced susceptibility to secondary West Nile virus (WNV) infection [4]. Additionally, the ISV Palm Creek virus decreased the replication of a WNV variant and Murray Valley encephalitis virus [5], and the ISV Nhumirim virus reduced the replication of Japanese encephalitis virus (JEV), St Louis encephalitis virus and WNV viruses in co-infected *Aedes albopictus* mosquito cells [6]. In the past decade, efforts to control *Culicoides* have mainly focused on modifying their habitat or using chemicals like topical repellents, adulticides and impregnated cattle ear tags [7]. Whilst these strategies offer some protection, they are often logistically challenging and not always very effective. In the future, combining these existing control methods with emerging symbiont-based strategies such as *Wolbachia* and ISVs could be advantageous for the control of *Culicoides*-transmitted arboviruses. *Wolbachia*, a bacterial endosymbiont, has emerged as a promising tool in controlling mosquito-borne diseases, particularly for combatting the spread of arboviruses like dengue, zika and chikungunya [8,11]. *Wolbachia* is maternally transmitted and can manipulate host reproduction through cytoplasmic incompatibility, leading to population suppression [12,13]. Additionally, *Wolbachia* reduces replication of some viruses in mosquitoes preventing the transmission of pathogens to humans [9,11, 14]. *Wolbachia* infections have been reported in several *Culicoides* spp. in Japan [15], Spain [16], Australia [17] and the USA [18], but none have been detected in the UK [19]. These studies have consistently found low-density *Wolbachia* infections in *Culicoides* hosts, which are often undetectable via conventional PCR methods. The use of more sensitive techniques such as quantitative PCR (qPCR), nested PCR and long PCR is suggested to identify these low-density infections [18].

In recent years, advances in metatranscriptomics, metagenomics and bioinformatics pipelines have enhanced the identification of viruses in haematophagous arthropods that may have been overlooked by surveillance measures. There have been several studies identifying novel viruses from field-collected *Culicoides* from Southern Japan [20], Yunnan, China [21], Thrace, Greece [22], and from some parts of Europe [23] and Mexico [24]. A comparative analysis between members of the Culicidae family has identified several novel viruses of the *Orthobunyavirus*, *Narnavirus* and *Iflavirus* genera within *Culicoides* assemblies [25], which indicate unique virome profiles of *Culicoides*. A study on different *Culicoides* spp., including *Culicoides brevitarsis* Kieffer (Diptera: Ceratopogonidae) from Yunnan, China, detected 3199 viruses across 5 orders and 12 viral families [26]. This screen identified six arboviruses of interest: Banna virus, JEV, AKAV, BTV, EHDV and Tibetan circovirus. However, as different *Culicoides* spp. were pooled, the information on the unique virome composition of *C. brevitarsis* was lost.

Within Australia, the *Culicoides* fauna is extensive, diverse and rapidly evolving due to the long-distance wind dispersal from neighbouring countries [27,28]. Whilst four Australian *Culicoides* species from *Avaritia* subgenus, namely *Culicoides actoni*, *Culicoides fulvus*, *Culicoides wadai* and *C. brevitarsis*, are known vectors for BTV, *C. brevitarsis* is the most widespread and prevalent across Australia, with distribution covering northern, central and eastern Australia [29]. This species has been found to drive the endemic spread of two BTV serotypes (BTV-1 and BTV-21) along the Australian east coast. Whilst the occurrence of bluetongue disease in Australia is an exceptionally rare event, where BTV does cause disease, mortality varies by serotype and can range from 20–70%, and outbreaks of BTV can cause large economic losses due to restrictions on animal movement and trade [30]. Besides BTV transmission, this species also transmits several arboviruses of economic importance and is responsible for Akabane epizootics [31] and several outbreaks of bovine ephemeral fever [32] in Australia.

To date, there are no published high-throughput metatranscriptomics studies on Australian *C. brevitarsis*, nor for any other Australian *Culicoides* spp. to explore their unique microbial diversity. Here, we used high-throughput datasets to explore the virome of *C. brevitarsis*, including arboviruses and insect-specific viruses present in localised field-collected samples from Australia. In addition, we also screened the population of *C. brevitarsis* for natural infection of *Wolbachia*. These data can be used to inform interventions such as *Wolbachia* and ISV-based superinfection exclusion to curb *Culicoides*-borne disease in Australia.

## Methods

### Sample collection, preparation and genomic sequencing

*Culicoides* spp. were collected overnight in the Australian summer (March 2024) from a region important for cattle rearing and beef production, Casino, New South Wales (NSW), using ultraviolet light traps and collection bottles containing 100% ethanol. Collection bottles were transported to the Commonwealth Scientific and Industrial Research Organisation (CSIRO) Australian Centre for Disease Preparedness (ACDP), Geelong, Victoria, on dry ice and morphologically sorted by species and biological sex [1]. COI sequences were assembled from next generation sequencing data to verify host species in a separate study [33]. Three replicates, each comprising a pool of ten females of *C. brevitarsis*, were used for downstream molecular works. Samples were homogenised in PBS with five 2.3-mm chrome steel beads (Cat no. 11079123 c, Daintree Scientific, Tasmania, Australia) using a Tissue Lyser (Qiagen, MD, USA) at 25 Hz for 2 min. Total RNA was extracted using Qiagen RNeasy Plus Mini Kit (Qiagen, USA). The quantity and quality of the yields were measured on a NanoDrop spectrophotometer (Thermo Fisher Scientific, MA, USA) and MultiNA Microchip Electrophoresis System (Shimadzu Biotech, Victoria, Australia), respectively. Each sample contained at least 1 ug of total RNA, which was dissolved in RNA stabilisation tubes (Genewiz, Suzhou, China). The samples were transferred to Genewiz (now Azenta, MA, USA) for rRNA depletion, strand-specific library preparation and sequencing with 150 bp pair-end reads with over 50 million reads produced for each sample. For *Wolbachia* detection, DNA was extracted in triplicates, each containing a pool of ten female individuals from the same field collection using Qiagen Genomic Tip 20/G kit (Qiagen, USA), following user-developed ‘isolation of genomic DNA from mosquitoes or other insects using the QIAGEN genomic-tip’ protocol available from QIAGEN (QG06.doc Sep-01).

### *Wolbachia* detection

To detect *Wolbachia* within the *C. brevitarsis* genomic DNA samples, a qPCR assay was carried out amplifying the 16S rRNA gene of *Wolbachia* (F: 5′-CATACCTATTCGAAGGGATAG-3′, R: 5′-TTGCGGGACTTAACCCAACA-3′) on QuantStudio 6 (Thermo Fisher Scientific, MA, USA) [34]. Reactions were run in a total volume of 20 µl, having 10 µl Luna Universal qPCR Master Mix (New England Biolabs, Victoria, Australia), 0.5 µl each of 2.5 µM forward and reverse primer and 2 µl of genomic DNA. As a positive control for the assay, we included DNA from *w*AlbB *Wolbachia*; *Wolbachia*-free DNA from *Aedes notoscriptus* was the negative control. The reaction condition for the assay was initial denaturation for 1 min at 95 °C followed by 45 cycles of denaturation for 15 s at 95 °C, 15 s at 55 °C and 15 s at 69 °C. The amplification was conducted for 1 min at 95 °C followed by 45 cycles of 15 s at 95 °C and 30 s at 60 °C, acquiring on the SYBR green channel at the end of each step, and run on QuantStudio 6 (Thermo Fisher Scientific, MA, USA).

### Data analysis

The initial quality control of the raw transcriptome dataset included using FastQC (v. 0.12.1) [35] and MultiQC (v. 1.11) [36] for generating quality reports. Adapter trimming was performed using TrimGalor (v. 0.6.6) [37]. The cleaned reads from each of the paired-end libraries were assembled to scaffold level using *de novo* assembler Spades (v.3.15.2) [38,39] with the *rnaviralSPAdes* parameter, and *K*-mers of 21, 33, 55 and 77 and ‘careful’ parameter turned on, to reduce mismatches and short indels. To check assembly quality, individual assemblies were also conducted for each replicate. Assembled contigs were also subject to a Basic Local Alignment Search Tool for translated nucleotides (blastx) search against the non-redundant (nr) protein database, a Basic Local Alignment Search Tool for Proteins (blastp) search against the nr viral protein database, as well as the viral RdRP database (all downloaded 1/3/2024) in diamond (v. 2.0.15) to investigate the presence of viral genomes. As part of the bioinformatic workflow, we also blasted our assembled contigs against the recently published Australian Biosecurity Genomics Database of notifiable diseases for awareness of viruses that could cause a nationally notifiable disease, although the sensitivity and specificity of this pilot database needs further work [40].

### Genome organisation

Assembled contigs with matches to RNA viruses were scrutinised further by extracting open reading frames (ORF) >100 bp and then translating them into amino acid sequences. The amino acid sequences were subjected to blastp searches against the nr protein database (09/07/2024). Sequences were then compared against the conserved domain database (CDD) (09/07/2024) to predict domain position. For the visualisation of genome organisation, R packages, ggplot2 (v.3.5.1) [41] and gggenes (v.0.5.1) [42] were used.

### Phylogenetic analysis

Alignments were created for each viral family using MAFFT (v. 7.490) [43]; due to the high number of species in the family *Rhabdoviridae*, the subfamily *Alpharhabdovirinae* was used instead for clear visualisation. Each alignment was then pruned using TrimAl (v1.4.22) [44] with the automated parameter. The amino acid substitution models were then determined using IQ-TREE (v2.3.5) [45]. Maximum likelihood trees were then constructed with 1000 bootstraps using IQ-TREE (v2.3.5) [45]. Further details of the determined model can be found in the figure captions.

### Virus nomenclature

Each novel virus that had complete or near-complete genome coding regions was named after the host that it was detected in, in this case, *C. brevitarsis*, followed by the genus they were taxonomically placed in by phylogenetic analysis. Finally, a numerical value was chosen to signify the chronological order that each virus species was detected, e.g. the one novel species of the family *Rhabdoviridae*, the genus *Ohlsrhavirus* was named Culicoides brevitarsis ohlsrhavirus 1. As exceptions, if a virus was shown to belong to a novel virus or if it was unclear which genus the virus belonged to, the virus was named, e.g. Culicoides brevitarsis peribunya-like virus 1 or Culicoides brevitarsis chu-like virus 1.

## Results

**Fig. 1.**
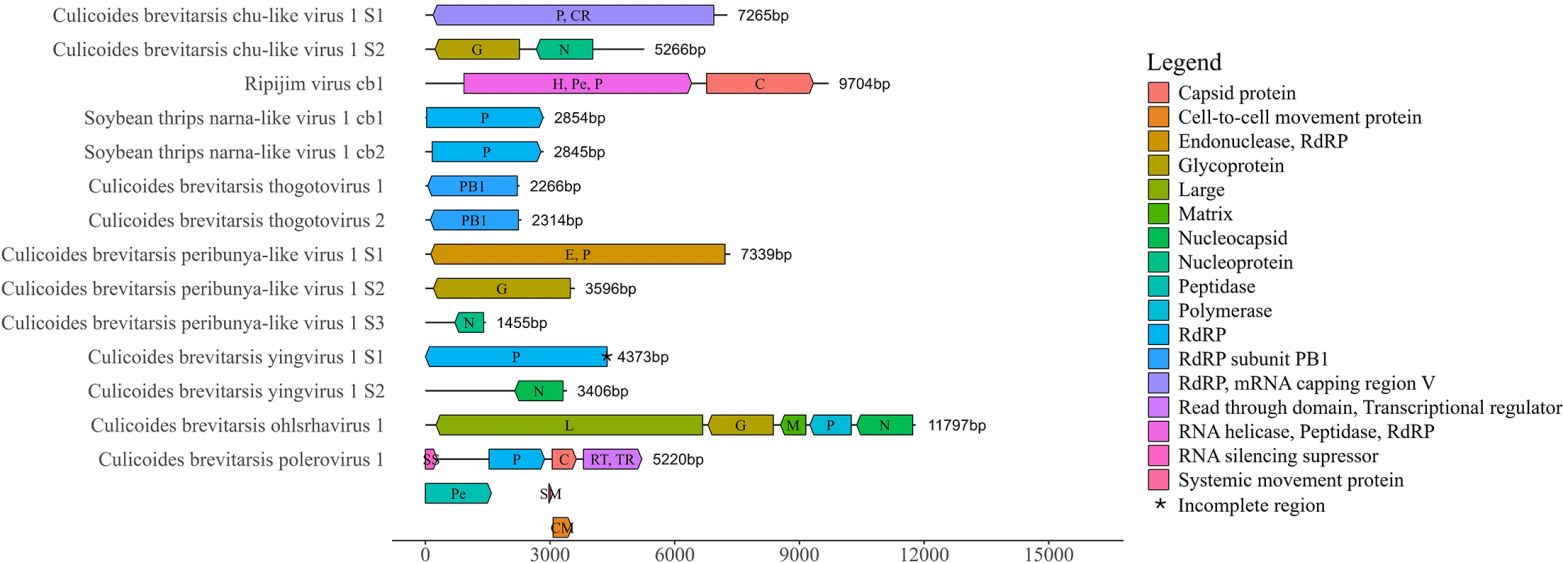
Genome organisation charts of assembled virus segments in this study. The protein domain positions of 14 assembled virus segments belonging to 10 viruses with complete or near-complete coding regions were predicted by comparing each assembled viral contig to the CDD. The unit of the *X*-axis is in bp. The abbreviations S1, S2 and S3 correspond to each segment where a virus was segmented. Incomplete coding regions are indicated by an asterisk.

Total RNA generated >50M reads per library that consisted of three replicates with ten *C. brevitarsis* individuals in each. After filtering against the host genome of *C. brevitarsis* (National Center for Biotechnology Information (NCBI) RefSeq accession: GCF_036172545.1), only 6–8% of reads were left unmapped. The remaining unmapped reads were then used to construct a Spades-based viral transcriptome assembly, resulting in an assembly of 36.89 Mb in size, with 76150 contigs, the largest being 11797 bp and an average length of 484.39 bp per contig. blast searches identified 6,889 hits to the nr viral protein database and 101 hits to the viral RdRP database. After combining both hit lists and applying stringent criteria (filtering out DNA virus hits and contigs <350 bp in length) and checking genome organisation, we finally identified 33 highly confident RNA viral contigs (Fig. 1). Twenty-two of the contigs belonged to 10 viruses with complete/near-complete coding regions; the remaining 11 contigs were fragments of three viruses (Table 1). We also screened C. *brevitarsis* samples for the presence of natural *Wolbachia* infection. All tested samples were found to be negative for *Wolbachia* infection.

**Table 1.** Putative viruses detected in *C. brevitarsis* transcriptomes with complete or near-complete genomes

Accession no. of detected viruses	Virus name	Order	Family	Genus	Genome type	No. of segments	Length of segment containing RdRP	Lengths of other segments	Closest blastp match (to RdRP segment)	Query cover to closest match (%)	Identity to closest match (%)	Accession no. of closest match in nr protein database
OZ194625	Culicoides brevitarsis ohlsrhavirus 1	*Mononegavirales*	*Rhabdoviridae*	*Ohlsrhavirus*	(−)ssRNA	1	11797	n/a	Culex rhabdo-like virus	97	48.95	WVL03141
OZ197699	Ripijim virus cb1	*Picornavirales*	*Dicistroviridae*	*Cripavirus*	(+)ssRNA	1	9704	n/a	Ripijim virus	100	96.39	WZH59379
OZ197702	Culicoides brevitarsis polerovirus 1	*Sobelivirales*	*Solemoviridae*	*Polerovirus*	(+)ssRNA	1	5220	n/a	Sugarcane yellow leaf virus	99	70.75	ASM94398
Segment 1 RdRP: OZ194618Segment 2: OZ194619	Culicoides brevitarsis chu-like virus 1	*Jingchuvirales*	*Chuviridae*	Unclassified	(−)ssRNA	2	7265	5266	Turkana Chu-like virus	97	43.26	UCW41650
Segment 1 RdRP:OZ194622Segment 2: OZ194623Segment 3: OZ194624	Culicoides brevitarsis peribunya-like virus 1	*Bunyavirales*	*Peribunyaviridae*	Unclassified	(−)ssRNA	3	7339	3596, 1455	Buffalo Bayou virus	91	33.11	QFQ60707
OZ197700	Soybean thrips narna-like virus one cb1	*Wolframvirales*	*Narnaviridae*	Unclassified	(+)ssRNA	1	2854	n/a	Soybean thrips narna-like virus 1	95	58.66	QQP18719
OZ197701	Soybean thrips narna-like virus one cb2	*Wolframvirales*	*Narnaviridae*	Unclassified	(+)ssRNA	1	2845	n/a	Soybean thrips narna-like virus 1	96	58.69	QQP18719
Segment 1 RdRP: OZ194626Segment 2: OZ194627	Culicoides brevitarsis yingvirus 1	*Muvirales*	*Qinviridae*	*Yingvirus*	(−)ssRNA	2	4373	3406	Hubei qinvirus-like virus 1	89	31.13	YP_009337847
OZ194620	Culicoides brevitarsis thogotovirus 1	*Articulavirales*	*Orthomyxoviridae*	*Thogotovirus*	(−)ssRNA	n/a	2266	n/a	Orthomyxoviridae sp.	99	83.68	UBB38850
OZ194621	Culicoides brevitarsis thogotovirus 2	*Articulavirales*	*Orthomyxoviridae*	*Thogotovirus*	(−)ssRNA	n/a	2314	n/a	Xiangshan orthomyxo-like virus	98	62.36	UDL13962

### Chuviridae

**Fig. 2.**
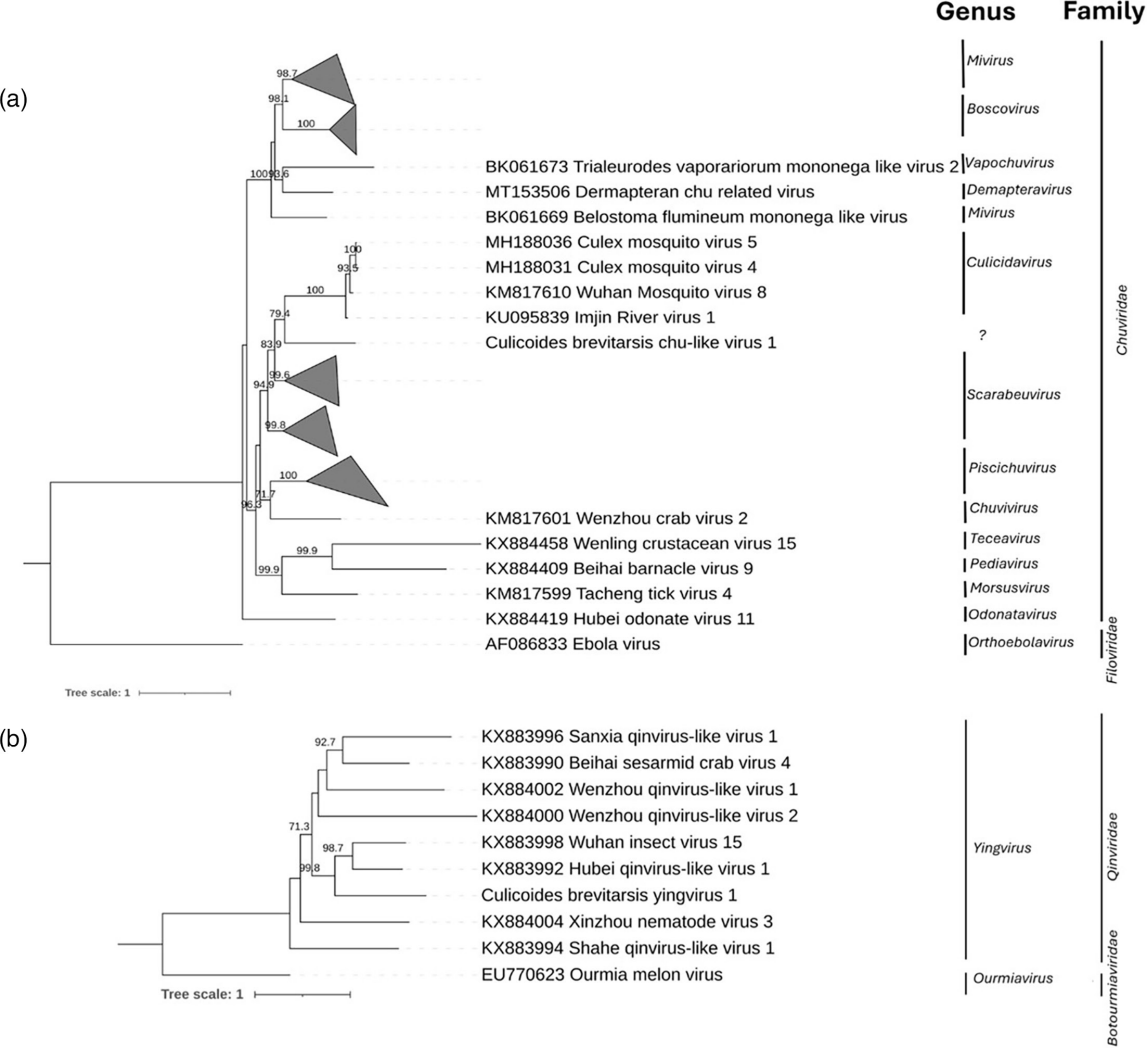
Phylogenetic trees for *Chuviridae* and *Qinviridae*. Maximum likelihood phylogenetic trees based on MAFFT alignments of the conserved RNA-dependent RNA polymerase and RdRP gene region of (a) *Chuviridae* using the LG+F+I+G4 model and (b) *Qinviridae* using the Q.pfam+F+I+G4 model. The outgroup for (a) is Ebola virus and for (b) is Ourmia melon virus. The support values are presented at the node based on 1000 bootstraps. The scale bars represent the number of amino acid substitutions per site.

One novel virus identified in this study belonged to the family *Chuviridae* and was assembled with both UTRs and a complete coding region. This was found to be closely related to the genera *Culicidavirus* and *Doliuvirus*, within the *Chuviridae* (Fig. 2, Table 1). Specifically, this detection consisted of two segments and CDD searches confirmed the presence of the RdRP, mRNA-capping region, glycoprotein and nucleoprotein, suggesting a complete or near-complete genome (Fig. 1). A blastp search of the ORF containing the RdRP region revealed the closest match in the nr protein database was the Turkana Chu-like virus with 97% amino acid query cover and 43.26% amino acid identity. The host range of the Turkana Chu-like virus is unknown, but members of the genus *Culicidavirus* are usually found in *Culex* mosquitoes, and the genus *Doliuvirus* has been found in the mosquito, *Culiseta minnesotae* [46]. According to demarcation criteria set by the International Committee on Taxonomy of Viruses (ICTV) for *Chuviridae*, a new genus is indicated by an amino acid sequence identity <31% in the L protein, and a new species is indicated by an amino acid sequence identity <90%; therefore, the virus we identified should be considered as a new species but not a new genus [46]. This virus had >31% amino acid identity to both genera *Culicidavirus* and *Doliuvirus*; therefore, it was not formally aligned to a genus, so we have tentatively named the virus Culicoides brevitarsis chu-like virus 1.

### Dicistroviridae

One genome identified in this study belonged to the family *Dicistroviridae*, genus *Cripavirus*, and was assembled with both UTRs and complete coding regions (Fig. S1, available in the online Supplementary Material, Table 1). CDD searches confirmed the presence of three major structural proteins (VP1, VP2 and VP3), an additional structural protein VP4 (N-terminal extension of VP3, cleaved from precursor VP0) and non-structural proteins (RNA helicase, peptidase and RdRP), suggesting a complete or near-complete genome (Fig. 1). A blastp search of the ORF containing the RdRP region revealed the closest match in the nr protein database was Ripijim virus with 100% amino acid query cover and 96.39% amino acid identity. The host range of Ripijim virus is unknown, but members of the genus *Cripavirus* have been detected in insects such as *Anopheles gambiae*, *Apis mellifera*, *Drosophila melanogaster* and *Nilaparvata lugens* amongst others and in vertebrates, one in a goose faecal sample that is possibly a result of ingesting insects [47]. According to demarcation criteria set by the ICTV for the genus *Cripavirus*, a new species is indicated by an amino acid sequence identity <90%; therefore, this should be considered the same species as Ripijim virus [47].

### Narnaviridae

Two genomes identified in this study belonged to the family *Narnaviridae* and an unclassified genus and were assembled with both UTRs and complete coding regions (Fig. S2, Table 1). CDD searches confirmed the presence of the RdRP in both sequences suggesting a complete or near-complete genome (Fig. 1). A blastp search of the ORFs containing the RdRP region revealed the closest match in the nr protein database to both sequences was the soybean thrips narna-like virus 1 with 95 and 96% amino acid query cover, and 58.66 and 58.69% amino acid identity. The host range of soybean thrips narna-like virus 1 is unknown but was isolated from soybean thrips collected in the USA, but members of the genus *Narnavirus* are generally considered viruses of fungi [48]. According to demarcation criteria set by the ICTV for the genus *Narnavirus*, a new species is indicated by an amino acid sequence identity <50%; therefore, these two viruses should be considered the same species as soybean thrips narna-like virus 1.

### Orthomyxoviridae

**Fig. 3.**
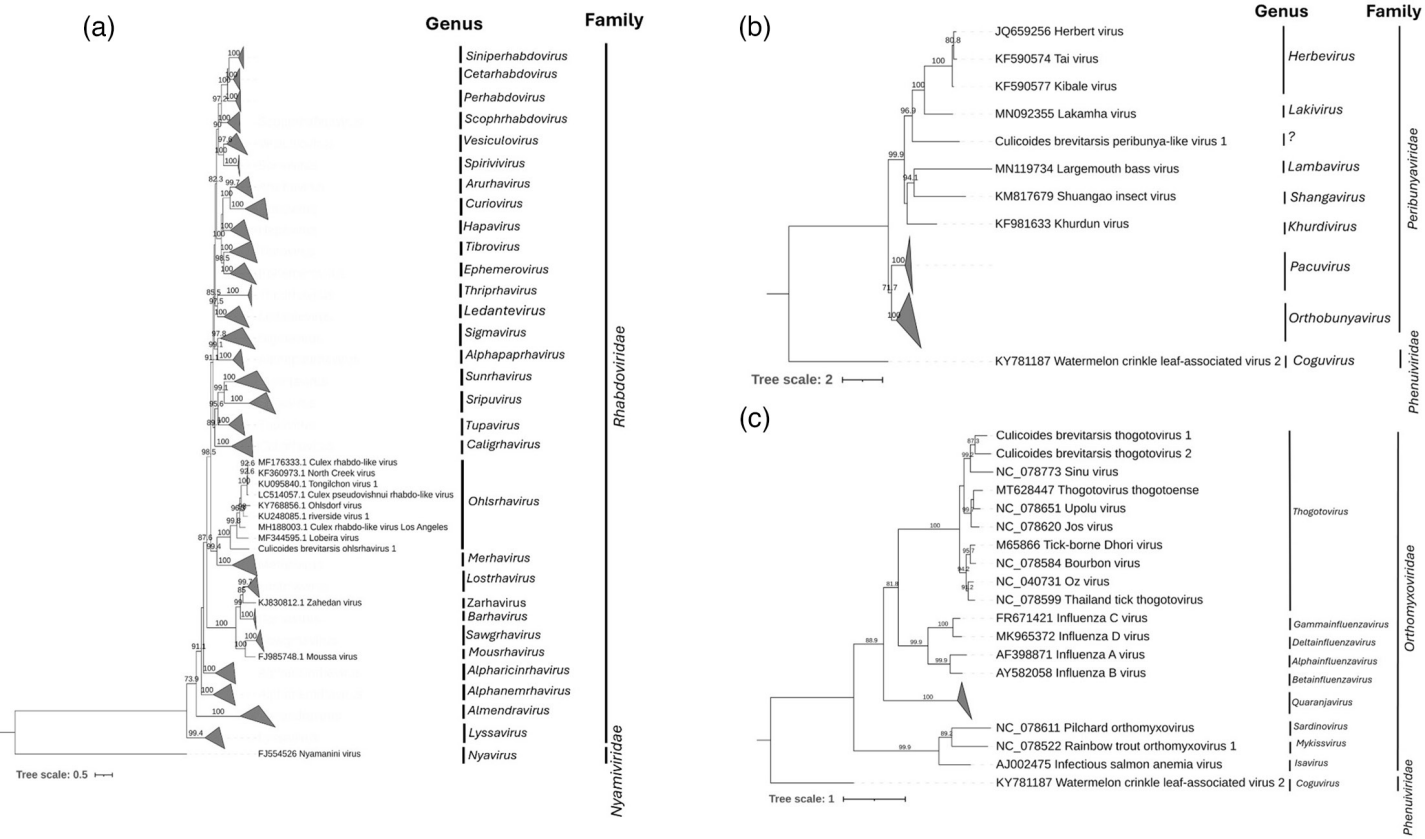
Phylogenetic trees for *Rhabdoviridae, Peribunyaviridae* and *Orthomyxoviridae*. Maximum likelihood phylogenetic trees based on MAFFT alignments of the conserved RNA-dependent RNA polymerase and RdRP gene region of (a) *Rhabdoviridae* using the Q.yeast+I+G4 model, (**b)** *Peribunyaviridae* using the Q.yeast+F+I+G4 model and (c) *Orthomyxoviridae* using the LG+I+G4 model. The outgroup for (a) is Nyaminini virus and for (b) and (c) is watermelon crinkle leaf-associated virus 2. The support values are presented at the node based on 1000 bootstraps. The scale bars represent the number of amino acid substitutions per site.

Two genomes identified in this study belonged to the family *Orthomyxoviridae* and the genus *Thogotovirus* and were assembled with five segments each with UTRs and complete coding regions for all but one segment which had a partial coding region (Fig. 3, Table 1). Eight segments could not be aligned to each of the two different RdRP segments (File S1). CDD searches confirmed the presence of RdRP subunit Polymerase Basic 1 (PB1), Polymerase Basic 2 (PB2), polymerase acidic and nucleoprotein for both viruses, as well as one segment containing a glycoprotein and one segment containing a matrix protein suggesting near-complete genomes (Fig. 1). A blastp search of the ORFs containing the RdRP subunit PB1 region revealed the closest match in the nr protein database was the unclassified virus named *Orthomyxoviridae* sp., with 99% amino acid query cover and 83.68% amino acid identity, and the Xiangshan orthomyxo-like virus with 98% amino acid query cover and 62.36% amino acid identity. The host range of the virus *Orthomyxoviridae* sp. is unknown but was isolated from *A. notoscriptus* collected in Brisbane, Australia [49]. The host range of the Xiangshan orthomyxo-like virus is unknown and was isolated from a pool of wild insect pollinators collected in Beijing, China [50]. Members of the genus *Thogotovirus* have been shown to transmit to humans by ticks and have been isolated from ticks such as *Boophilus* sp., *Rhipicephalus* sp., *Amblyomma* sp. and *Hyalomma* sp. but also mosquitoes. Serological evidence suggests that cattle, sheep, goats, donkeys, camels, buffaloes, rats and waterfowl are also susceptible [51]. No numerical demarcation values have been established by the ICTV; however, confirmed species of the genus *Thogotovirus* have RdRP PB1 amino acid identities between 61.06 and 96.85%. Therefore, as our two virus sequences have 83.68 and 62.36% amino acid identities to their closest matches, and 60.47% amino acid identity to each other, these two viruses should be considered novel species. We named these Culicoides brevitarsis thogotovirus 1 and 2.

### Peribunyaviridae

One genome identified in this study belonged to an unnamed genus within the family *Peribunyaviridae* and was assembled with three segments with UTRs and complete coding regions (Fig. 3, Table 1). CDD searches confirmed the presence of the RdRP, endonuclease, glycoprotein and nucleocapsid protein, suggesting a complete or near-complete genome (Fig. 1). A blastp search of the ORF containing the RdRP region revealed that the closest match in the nr protein database was the Buffalo Bayou virus, with 91% amino acid query cover and 33.11% amino acid identity. The host range of Buffalo Bayou virus is unknown but was isolated from a dead blue jay (*Cyanocitta cristata*) bird, within its decomposing brain with fly larvae present, collected in Houston, USA [52]. The host ranges of the family *Peribunyaviridae* can be restricted, but some have been found in humans, other mammals, birds, mosquitoes, midges and sandflies [53]. According to demarcation criteria set by the ICTV for the family *Peribunyaviridae*, the inclusion in a genus can be defined by forming a monophyletic clade with other viruses in the genus using the L protein sequence; therefore, as it is part of a non-monophyletic group, this should be considered a new genus within the *Peribunyaviridae* [53]. We name this virus Culicoides brevitarsis peribunya-like virus 1.

### Qinviridae

A near-complete coding genome with UTR was also identified in this study belonging to the family *Qinviridae*, genus *Yingvirus* (Fig. 2, Table 1). No putative conserved domains were detected in a CDD search (Fig. 1). A blastp search of the ORF containing the RdRP region revealed that the closest match in the nr protein database was the Hubei qinvirus-like virus 1 with 89% query cover and 31.13% amino acid identity (Table 1). The host range of Hubei qinvirus-like virus 1 is unknown and was isolated from a pool of mixed dipteran insects including *Drosophila*, *Episyrphus*, *Sarcophaga*, *Muscina* and *Ptecticus* spp. collected in Hubei, China [54]. More generally, members of the genus *Yingvirus* have been found in decapod crustaceans, blattodean and dipteran insects, gastropods and nematodes [55]. According to demarcation criteria set by the ICTV for the genus *Yingvirus*, a new species is phylogenetically distinct based upon analysis of the RdRP as no numerical demarcation values have been established; therefore, this should be considered a novel species [55]. We named this virus Culicoides brevitarsis yingvirus 1.

### Rhabdoviridae

One genome identified in this study belonged to the family *Rhabdoviridae,* genus *Ohlsrhavirus*, and was assembled with both UTRs and complete coding regions (Fig. 3, Table 1). CDD searches confirmed the presence of the RdRP, mRNA-capping region V, glycoprotein and nucleocapsid protein, suggesting a complete or near-complete genome. A blastp search of the ORF containing the RdRP region revealed that the closest match in the nr protein database was the *Culex* rhabdo-like virus with 97% amino acid query cover and 48.95% amino acid identity. The host range of the *Culex* rhabdo-like virus is unknown but was isolated from *Culex* mosquitoes collected in Western Australia and later detected in *Culex australicus* also collected in Western Australia [56,57]. Whilst members of the relatively new genus *Ohlsrhavirus* generally infect insects and have been found in culicine mosquitoes, other members of the family *Rhabdoviridae* can infect humans, marsupials, ruminants, rodents, insects, fish or plants [58]. According to demarcation criteria set by the ICTV, a new species is indicated by an amino acid sequence identity of <90% in the N and L proteins and <85% in the G proteins [58]. Additionally, blastp searches revealed the N protein had 90% amino acid query cover and 33.82% amino acid identity, and the G protein had 80% amino acid query cover and 45.88% amino acid identity to the closest match; therefore, this virus should be considered a new species. We named this virus Culicoides brevitarsis ohlsrhavirus 1.

### Solemoviridae

**Fig. 4.**
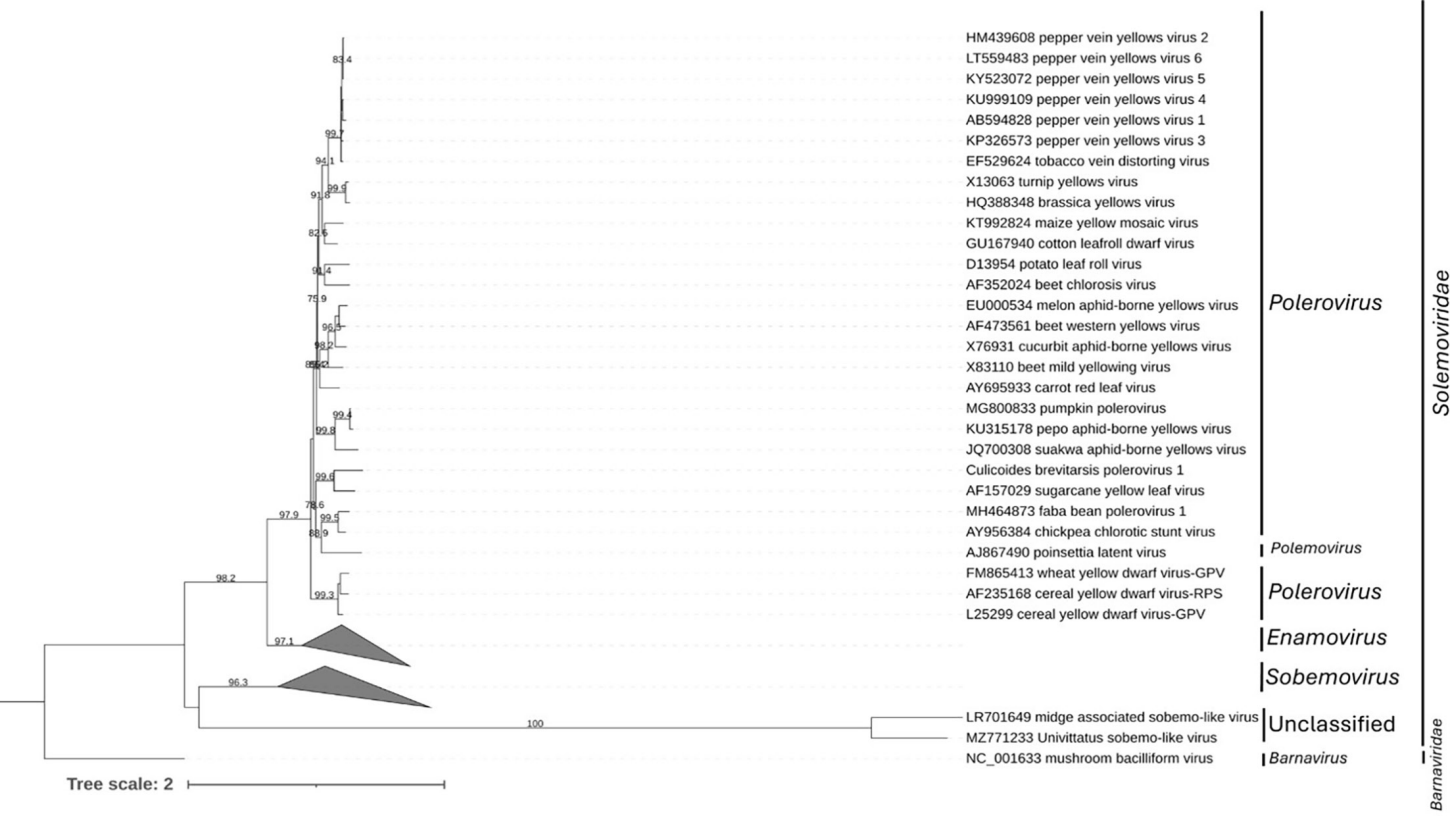
Phylogenetic trees for *Solemoviridae*. Maximum likelihood phylogenetic trees based on MAFFT alignments of the conserved RNA-dependent RNA polymerase and RdRP gene region of *Solemoviridae* using the LG+I+G4 model. The outgroup is the mushroom bacilliform virus. The support values are presented at the node based on 1000 bootstraps. The scale bars represent the number of amino acid substitutions per site.

One genome identified in this study belonged to the family *Solemoviridae*, genus *Polerovirus*, and was assembled with both UTRs and complete coding regions (Fig. 4, Table 1). CDD searches confirmed the presence of the RdRP, peptidase, coat protein and readthrough protein, suggesting a complete or near-complete genome. A blastp search of the ORF containing the RdRP region revealed that the closest match in the nr protein database was the sugarcane yellow leaf virus with 99% amino acid query cover and 70.75% amino acid identity. The host range of sugarcane yellow leaf virus includes sugarcane (*Saccharam* and *Erianthus* spp.) as well as grain sorghum (*Sorghum bicolor*) and Columbus grass (*Sorghum almum*) and may be transmitted by *Melanaphis sacchari* and *Rhopalosiphum maidis* aphids [57,59]. More generally, the genus *Polerovirus* infects plants and is transmitted by aphid vectors [60]. According to demarcation criteria set by the ICTV for the genus *Polerovirus*, a new species is indicated by an amino acid sequence identity <90%; therefore, this should be considered a new species of *Polerovirus* [60]. We named this Culicoides brevitarsis polerovirus 1.

### Fragments: *Phasmaviridae*, mycovirus and *Endornaviridae*

In addition to the complete and near-complete virus genomes, three virus fragments matched to the genus *Orthophasmavirus* of the family *Phasmaviridae* in the nr protein database, containing a glycoprotein and ranging from 275 to 2,741 bp in length. One fragment of 909 bp matched to unclassified mycovirus in the nr protein database. Seven fragments matched to the family *Endornaviridae* ranging from 382 to 618 bp in length in the nr protein database (File S1).

## Discussion

*Culicoides* spp. are important vectors for many viruses important to animal and human health; yet to our knowledge, no high-throughput virome studies of any *Culicoides* spp. have been conducted in Australia, including *C. brevitarsis*, a major vector of BTV. Furthermore, no study has specifically investigated the virome of solely *C. brevitarsis* or investigated *C. brevitarsis* for the presence of *Wolbachia* which can influence insect viromes. The viromes of these *Culicoides* spp. are important to investigate especially for novel and emerging pathogens as they can potentially impact human and livestock health and the economy, but also for insect-specific viruses which can impact transmission of these pathogens.

Here, we present the first comprehensive analysis of the virome of a *Culicoides* sp. in Australia, *C. brevitarsis*, investigating and expanding the diversity of viruses in *Culicoides* spp. Our data revealed the presence of 10 complete or near-complete viruses, as well as 3 fragmented viruses, belonging to a total of 11 virus families, in samples from Casino, NSW. Of the complete or near-complete viruses, seven of these are likely novel virus species, with one belonging to a novel genus. Given that the majority of virus families found in this study have been previously found in other *Culicoides* spp. (*Chuviridae*, *Dicistroviridae*, *Narnaviridae*, *Orthomyxoviridae*, *Peribunyaviridae*, *Phasmaviridae* and *Rhabdoviridae*) or other closely related insects (*Qinviridae* in *Culex* spp. mosquitoes), it is likely that these do actually infect *C. brevitarsis* [21,24,26, 61].

We also revealed the absence of any *Wolbachia* spp. in our sample using sensitive qPCR assays. However, testing additional samples from different locations in Casino, NSW, would help to confirm whether there is a possibility of low infection frequency or undetectable low-density prevalence of *Wolbachia*.

### *C. brevitarsis* is a possible vector of novel vertebrate arboviruses

Of the viruses identified in this study, some are closely related to human pathogens of global medical significance. A notable characteristic they share is their ability to be transmitted via arthropods. Among the viral groups represented, within the *Peribunyaviridae*, the closely related genus *Orthobunyaviru*s arguably has the most impact on humans and includes pathogens such as OROV [62], La Crosse virus and Californian encephalitic virus [63]. These viruses are primarily transmitted via haematophagous insects, notably mosquitoes and midges. In humans, infections typically result in symptoms such as fever or malaise; however, in rarer cases, infections with these viruses can lead to encephalitis or meningitis [64,65]. Recently, OROV has been associated with outbreaks in South America [66,68], with cases also detected in the USA [69,70]. OROV is predominantly transmitted by *Culicoides paraensis* [71]. In addition, several orthobunyaviruses are animal pathogens, with viruses of the Simbu serogroup responsible for disease outbreaks in birds and livestock [72,73]. As such, the detection of orthobunyaviruses in our samples is highly significant and may have implications for disease transmission by *Culicoides* in Australia. Furthermore, it raises concerns about the potential for *C. brevitarsis* to serve as a vector of exotic pathogens if they were introduced into the country.

Viruses with close homology to the *Thogotovirus* genus were also identified in our samples. This group of viruses has also been linked to human disease, and infections are associated with febrile disease and, in rare cases, can also lead to meningitis and encephalitis [74,75]. Members of this genus (e.g. Thogoto virus, Dhori virus and Bourbon virus) are generally transmitted by ticks but have also been detected in mosquitoes [76,78]. The detection of novel thogotoviruses in *C. brevitarsis* suggests that a wider variety of haematophagous insects may contribute to their transmission and require further investigation.

Previously, viruses of the family *Rhabdoviridae* have not been associated with being transmitted by midges, but laboratory experiments have shown they can be infected [79,80]. Recently, a relatively new genus of rhabdoviruses that has been discovered in mosquitoes globally, known as ohlsrhaviruses, was highlighted in our samples. Whilst novel rhabdoviruses have been isolated from midges more recently [81,82], the significance of the ohlsrhaviruses in *Culicoides* is not well understood. Whilst the ohlsrhaviruses have not been implicated in disease, Chandipura vesiculovirus (CHPV) (belonging to the *Vesiculovirus* genus within the *Rhabdoviridae* family) is the agent of a significant human disease that has caused sporadic outbreaks in India [83]. Symptoms include high fever, convulsion, unconsciousness, encephalitis and coma [84,86]. CHPV is transmitted by a variety of biting insects and as such demonstrates the potential that *Culicoides* may also play a role in the spread of some rhabdoviruses.

Although *Culicoides* are not major vectors of human disease like mosquitoes are, they are crucial carriers of arboviruses that affect livestock animals [3,87] and can have significant economic impacts [88]. Arguably, the most significant of these is the bluetongue virus (which infects ruminants) with a significant global impact [89,91]. However, many others including EHDV (ruminants), SBV (cattle, sheep, goats), AHSV (horses) [92,93], AKAV and others (bovine ephemeral virus, vesicular stomatitis virus and Palyam virus) have significant implications for animal health. Whereas comparison with the recently published Australian Biosecurity Genomic Database [40] did not reveal any reportable known arboviruses, the phylogenetic analysis of the novel species/genera detected in this study suggests that these novel viruses may infect vertebrates based on homology with existing arboviruses. Further experimental evidence is required using isolated viruses to test their host range. In addition to this, further understanding of the pathogenicity of these viruses and the vector competency of Australian *Culicoides* spp. and other potential vectors is important to understand the risk novel viruses pose to both human and animal health.

### Insect-specific viruses

Current knowledge on insect-specific viruses in *Culicoides* spp. is limited, and of the virus families, we found that members of the *Chuviridae*, *Dicistroviridae* and *Phasmaviridae* had previously been found in *Culicoides* spp. RNA-Seq datasets [22,25]. Our findings reveal additional insect-specific viral diversity including the presence of a novel member of the *Qinviridae*, a relatively newly discovered family of viruses, but also new species diversity within the family *Chuviridae*.

These four virus families have previously been detected in other biting insects as well as non-biting insects. Studies of the viromes of other biting midge genera are limited, but all four of these families have been shown to occur in *Culex*, *Aedes* and *Anopholes* mosquitoes [23]. In ticks, *Chuviridae*, *Dicistroviridae* and *Phasmaviridae* have been found, but members of the *Qinviridae* have not been detected [94,99]. All four of these families are also frequently found in other non-biting invertebrates at varying incidences, e.g. *Dicistroviridae* is commonly found in honey bees, but *Chuviridae*, *Phasmaviridae* and *Qinviridae* are not present [100]. Regardless of the incidence, these four families found in *C. brevitarsis* in this study predominantly infect insects, suggesting that they are likely to be ISVs.

ISV families can have a diverse effect on host fitness. Of the four ISV families found in *C. brevitarsis*, the host effects of *Dicistroviridae* in other insects are the most studied. Some dicistroviruses can have a highly pathogenic effect on the host, e.g. cricket paralysis virus can cause paralysis and death when cricket nymphs are infected orally [101]. Some can weaken the immune system and leave insects susceptible to other microbial infections, e.g. Israeli acute paralysis virus can weaken honey bee immunity and ultimately lead to colony collapse [102]. Others do not cause any obvious symptoms, e.g. Nilaparvata lugens C virus had no visible symptoms or differences in mortality between treatment and control groups when brown planthoppers and *Nilaparvata lugens* were infected orally [103]. Some ISVs also have the ability to cause superinfection exclusion, whereby a virus that establishes in a host first can result in the prevention of other viruses from replicating, but to our knowledge, evidence of this has not been observed in the four ISV families found in *C. brevitarsis*.

Currently, most studies of *Chuviridae*, *Phasmaviridae* and *Qinviridae* have focused on virus discovery, characterisation or evolutionary history studies, and so, their pathogenicity to their hosts and their ability to cause superinfection exclusion is currently unknown.

### Viruses of fungi and transient viruses

Some virus families including *Solemoviridae* as well as those families that our virus fragments matched to, *Endornaviridae*, *Narnaviridae* and *Mycovirus*, are known viruses of plants, fungi or both and are sometimes present in insects. The majority of *Solemoviridae* are pathogens of plants and can be transmitted by insects such as aphids by sap-feeding [104,105]. However, some unclassified *Solemoviridae* have also been found in *Culicoides* spp., and other insects suggesting they can also infect insects [24]. The *Solemoviridae* we found in *C. brevitarsis*, Culicoides brevitarsis polerovirus 1, was taxonomically placed in the genus *Polerovirus*, separate from the insect unclassified *Solemoviridae* suggesting it is possibly a plant pathogen. The closest relative to Culicoides brevitarsis polerovirus 1 was the sugarcane yellow leaf virus, one of the most widespread and damaging viruses to sugarcane worldwide. The presence of this potential plant pathogen could be due to a shared ecological interaction, e.g. feeding on a shared nectar source of infected plants, or from contact or consumption with the faecal substrate during larval development [106]. However, some viruses have been shown to use plants as an intermediate site, with no replication in the plant, to transmit the virus between insect hosts, e.g. Aphis citricidus picornavirus [106]. Therefore, whilst Culicoides brevitarsis polerovirus 1 shows potential to be a plant pathogen, it is important to first investigate its host range.

*Endornaviridae*, *Narnaviridae* and unclassified mycoviruses have not been found in other *Culicoides* spp. However, *Narnaviridae* has been found in other insects, e.g. Mosquito narna-like virus 1 in *Aedes* and *Armigeres* mosquitoes and barley aphid RNA virus 6 and 7 in the corn leaf aphid, *Rhopalosiphum maidis* [107,108]. Furthermore, *Endornaviridae*, *Narnaviridae*, unclassified mycoviruses and *Solemoviridae* are likely present as a result of consuming fungi/plants or the presence of fungi in the *C. brevitarsis* samples. Therefore, it is possible to consider these as transient viruses that do not infect *C. brevitarsis* itself.

### Concluding statements

In this study, we explored the virome of field-collected *C. brevitarsis* from Australia for the first time to reveal a diversity of novel and known viruses. The sample sizes used in this study were limited and consisted of females only; therefore, the true diversity of viruses is expected to be larger than those found in this study. Regardless, we were able to identify three novel viruses which were shown to be closely related to vertebrate arboviruses, two other novel viruses that were closely related to insect-specific viruses and one novel virus that was closely related to an important plant pathogen. Further characterisation focused on investigating host range and vector competency is necessary to understand the relationship between these novel viruses and their hosts and is important in improving our knowledge of potential emerging pathogens.

## supplementary material

10.1099/jgv.0.002076 Uncited Fig. S1.

## Abbreviations

AHSV: African horse sickness virus
AKAV: Akabane virus
BTV: bluetongue virus
CDD: conserved domain database
CHPV: *Chandipura vesiculovirus*
EHDV: epizootic haemorrhagic disease virus
ISVs: insect-specific viruses
JEV: Japanese encephalitis virus
NCBI: National Center for Biotechnology Information
nr: non-redundant
NSW: New South Wales
OROV: Oropouche virus
qPCR: quantitative PCR
SBV: Schmallenberg virus
WNV: West Nile virus
