# Supplementary material for: Characterisation of the virome of Culicoides brevitarsis Kieffer (Diptera: Ceratopogonidae), a vector of bluetongue virus in Australia
Source: J Gen Virol. 2025 Feb 20;106(2):002076. doi: 10.1099/jgv.0.002076 (PMC11842880; doi:10.1099/jgv.0.002076)
Supplement: Uncited Fig. S1. [file jgv-106-02076-s001.pdf]

Characterisation of the virome of *Culicoides brevitarsis* Kieffer (Diptera: Ceratopogonidae), a vector of Bluetongue virus in Australia

Stephen R Sharpe, Mukund Madhav, Melissa J Klein, Kim R Blasdell, Prasad N Paradkar, Stacey E Lynch, Debbie Eagles, Adam J. López-Denman, Khandaker Asif Ahmed

CSIRO Australian Centre for Disease Preparedness (ACDP), East Geelong, VIC 3220, Australia.

Email: stephen.sharpe@csiro.au, adam.lopez-denman@csiro.au, khandakerasif.ahmed@csiro.au

Supplementary figures

Fig S1. Phylogenetic tree for *Dicistroviridae*.

Maximum-likelihood phylogenetic tree based on MAFFT alignment of the conserved RNA-dependent RNA Polymerase, RdRP gene region of *Dicistroviridae* using the LG+I+G4 model. The detected virus is denoted by a blue asterisk. The outgroup is Acheta domesticus iflavirus. The support values are presented at the node based on 1000 bootstraps. The scale bar represents the number of amino acid substitutions per site.

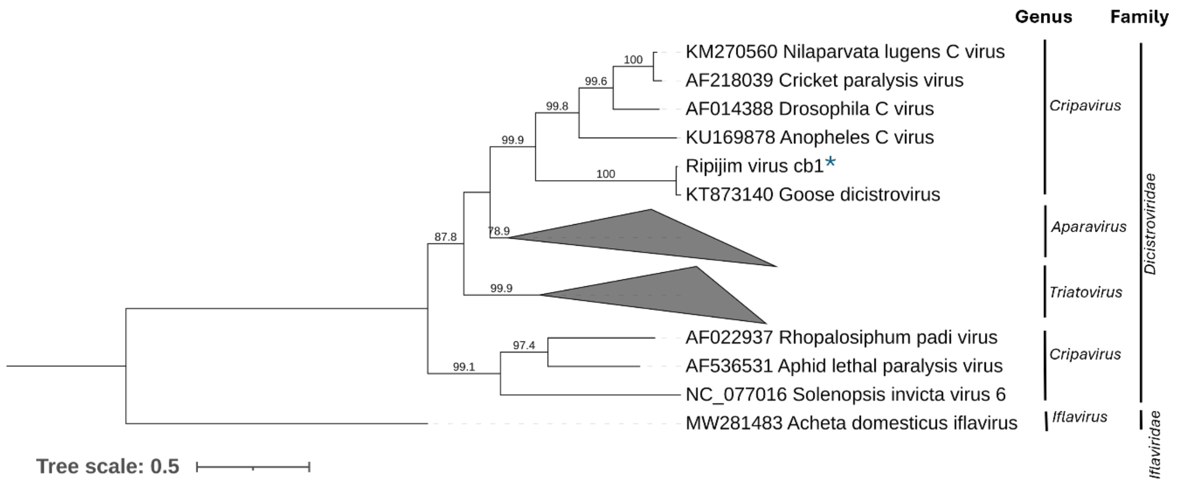

Fig S2. Phylogenetic tree for *Narnaviridae*.

Maximum-likelihood phylogenetic tree based on MAFFT alignment of the conserved RNA-dependent RNA Polymerase, RdRP gene region of *Narnaviridae* using the Q.pfam+G4 model. The detected viruses are denoted by a blue asterisk. The outgroup is Rhizoctonia mitovirus 1. The support values are presented at the node based on 1000 bootstraps. The scale bar represents the number of amino acid substitutions per site.

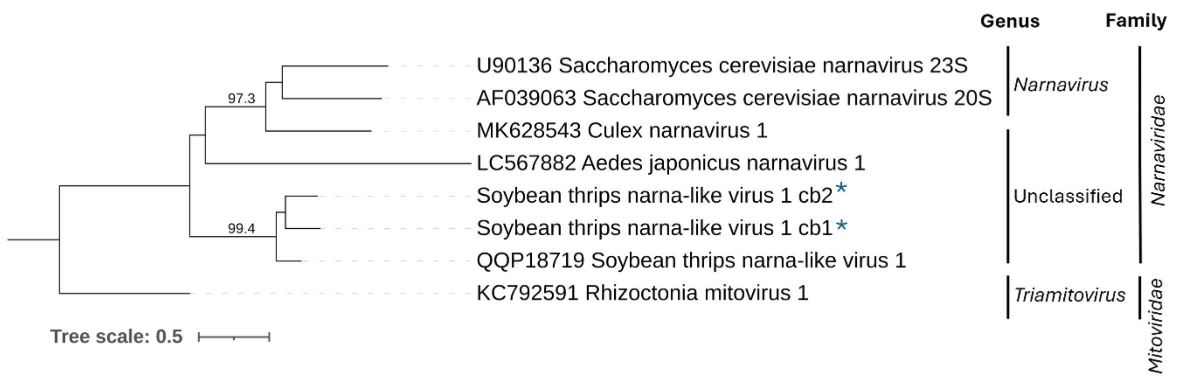

Supplementary data

>Putative Phasmaviridae fragment 1

GCAATTCTTCATTTTTGCAGAAAATATAATTTTGATTCTGAAAAACGTTTGGAGCATTATGCAA
CGGAATGCCAGCTTCGAGCCATAATGGCGCTACTACGTTCATGTTCGCCGTACTTCGAGC
TGTTTGAGAAATTGGAAAATGAATCAATTAATCCTTGGACATCCTTATTTTCACATTCAGATAG
TCCAAAAGTGAGACGAATGAAAGTTATAATTATTCGCATTTCCGGATTGGAGAAGGTGTTCC
GGAAATTATAAATCAGTAGCGACATCTGGTTTTCAAAAGTTAAATCAATAGTGAGTTTCAGAA
TATTTAATTTTTAAATTGCAAGAAAAAAAATCATAAAAATAACCATAATCATAAATCATAAAAATT
TGAATAACTAGTTTAGTAATTAGTAATGAAAATTATTTTTAGGAAAGATAAAATAAAATGTTTAAG
AAAAAAAATTAAGGACTCACTAAAATTAAAGCATTTTTTAACGGTTGTTTAAATTAGGTGTTGA
GTTGTTCTTTGAAACTGCCTATTTGTGCAATATAGAAACATGCATCGTTGAAGTAACATAACA
ATTGGTGCCAAGATGGAGGCGATACCCATGATCATCATACCACCATTTAAACTATCCATAA
AAGTTTTGCTTGAAAATATGGAAATTCCAGTGTTTACTCCACCTATGGCAACTGAACCTCTG
GAGTGTTGAAACGATACTTTGCCTTCAAAGATCGGTATGTTCTCAATGTGAAGTGTTATATTTT
TACTTGGTATGAAAATCATACAGTATTCCTCAAAATTTTCGTATTCTAGATAATAATATTCTGGT
CCGCAGCTTAAATAGGGTCTGGTAAAAGAACAGTTGCTTTTTATTTGCATTACTCCATCTCTA
TTTGCGATGGATTGGTACATGATGTATGGCATTTTAGTACATCCTATACAGGAGTACTTTTCTA
TAATATTTATTTCGCATTCTCCTGGTGCAGCTGCAGTGTGCGAAAATTTTAAATTACCAATTTT
CACCGGAATTTGGCCATTTGCCCTTTTTTTAACAATTACTGTGTTTCCTACTTTTTCGTATTTTT
CGACTGGTTTTAAGTCTCCTTTTTTAATTTTGTCACGCGTTATCTTTAATTGAGACACTGGCAC
TTCGCAGTGTACGTCACACGAAGCCACTAGACAATTTATATCAAATAATGGATAGTTTGTAGT
AGTGTATATAGGTTTTTGTCCCAAATTGAATTGGAAATCACCTACGAATTCAATCGCTGGGTG
ATCCAGCGGGCTTGCATGAACATGATAAAATACTTCCCTTTCATTAAGCAATGATTCTGTTAC
TCTTTGTTTTTCAAACGTTGGTTCTTGAAATTGCAATGAAATCTCTTTTGCAATATCCATGAATG
TCCGCAAAAAAAATGCAGGATTCGATGCAGTAACATACGAAGTGTATAAATGGTCGTTATAT
ACATATTCAATTGTTGCATACCACCCAGAAGATCTTTCTTTGTACACTATTGCTGAAGGACC
CGTGGGGACAGCATACACCGAGTAATGAACACAGCTTGATGTTCCATACCATGGACAAGT
TACGCCTTCACACTGAAGTGTTGTATCCCCACACCCGTACTTATGAACGTAATGTTCCTCTG
TAGTTTTGTTCACAAGCTTTGCCAACTTTGTATTTATATTGCATTTACCATCATAACAATCTGGA
TGACCTTTACATTGGACAGACGAATTTGTTCTTATGGTGTACTCGGATGTCTCATACATTGGA
ACATATCGATGATATTGTCCAAAATCTTTTAATTTGATGGTGAAGACTCTCTCTAAATTGTTAAA
GAAGGTTATTTGTTTACCAGATTCCAAGTCTATTTGAACTACAGGCACAGTATTGCAAACAC
CATCGTGTAACATACAAATTGCGCTGTCACTTTTAAGGTAAATCGCAATATCATTTGCCATAT
TTCCTCCAACACTCGAAGCAAGTAATCCAGTAACAAGAACTTTTTTTGCAATATTGTGTGAAA
GTTCGCAGCGCTTTCCTAGTACTTTTTTCCTGTAAGGCAAAGTCAATTCGTACTTATTTTCAA
CTTCGTATTTATCATTTCCTCCTCGACATCGACCTTTTATTTCATTTCTATCGCTTTTCAGTTGT
CTTTTATAGTTGAAATAATAAGCTATATGGTCTACGATGTATTTAATTTGCGGTTTGAAGCAGC
AAAAGAGCAACACAAAAAGTGAACCCAAGAGAAGCAGTGCGAGAGATGCAAACAACACT

GGCAAAAATATTTGTGTTTTTCTGCATTTTTCAAATAGCTCGTTGTAGGGACAAAATAATCCG
CATTCTGCACAATCTTCTTTGGAAATAATAGTCCCCGAGTCGAGATTCGAGCAGTTGTATAA
AATTTTTCCAAAATAAAGGTCTTCTAAAATTCCAGTTGCATTCTTGATTGTCTCAAATCCAACA
TGAATGTCACAAGAATACCTTGGGTCTACTTTTAATATGCCATTAGTAACAGCAATAATTGGC
TTAGCCAAGTGATGATCCATCTTGATGATAACTTGATGATCTCTTTGAATTCTTCCAAGCACTT
GTAAGCCGGTAAGTTGTCGATAAGTAACCAAATGCTCCTAGCTCAAAAAAAAAAACAGCAA
AAATATTAGTCAAAATTTCTTTTTGTGACAGTGGTGTGACAGTGTGAGTGACAGTGAGTGACA
GTGACAAAGTGTAAAATCTGAAAAT

>Putative Phasmaviridae fragment 2

AAAGGGCTTGAACATCCCATAAAACATTATTTATTGAATGGTAAAGAGCTTGTAACTAAGTTT
CCTACGAGTGCATCCGCACATGTTAAATTTGGCAACATTAATGTCGGTAAACTTCATTTGATT
CGCGGAAAATGCAACATAAATTTAGTTTACCAAAGTTCATGTTACGGCTGCAATACAAAACC
TTATTTGTTATTTCAAGCAAGCAATATAGAGAATGAAGGAGACATAGAATTTTCATCAAATTGC
TCTTTCGCGCATGATTATCTATCTTGCAATGCTGAAACATACAAATTAGAATACAAGACATTC
AAAAGCTATTGCTATATTTCTATTCTTGAGCTAAACCAAACAATATATATTGCTAATATTGAAAA
ATTTGAGGGAGAAATATCGTTCTCGACTCCACTTTCTTCAACTTCTGATTTTGAAAGTATCATG
AATGTTGTAAAAAGCAATACTTTTTATAAAACTGTCTATTCGACATTATCTGGCA

>Putative Phasmaviridae fragment 3

TTGTGGTCCAACAATTTTCATTACAAACACCGAGACGATATGTTTGGATAAAACGATCTGCA
AGCCGGTTAACATGGTTCAATTGTCATTGCGAAGCGGTGTTTTACAGACATTAGAGGATAAA
AATGGAGAAATATTGGAACTAAAAATTCTGGACATCGGTTTTTACTATACATATTATGAAGTGT
ATGAAACAACTGATTTTACATTAACGGTAAATAAACTCTTTCTTCATTTTAAAATTCTATTAGAC
TTTTGTTATTTTTAGGTTCCAACCCCAATTGCGACGTGTGATCTATCAGGAGAGTGCACACA
ACACACATGCATGCCTTCAACAAAACATCCAAGCTATAATAATGACAGCATAACAGATAAAT
TACATAAATATGGATGTGAGGTTTCTACATTGGGATGCGTAGACAAATATTGTGTTCTTTTTCC
CATCGTTTCGAGTCGTTGTATTCACTATAAGATGATTATAAATCCAACCGGACTTAGATCAAC
CATTTTTAAGTTATCAACAAGAGGTTTTTACG

>Putative mycovirus fragment 1

GTTGTCCTGGGCGCATGGCGGCACAGAGAAGTTTGTAACGCGCTTCGCTGCGTATGATAC
ATACTTCTCTATTGACTTTCCACGGTTTGACTCATCAATCGAACCTGAGTGGATCGCACACA
TTCTCTCTGTGTGCCGTACCGCTTTCGTTGGTGATGATGCGTCACGTGACATGTATTGGGA
GTTTGTCTTCCAATCCATAGTGCACGTACCAGTGGTCCTCGGAGATGGGGTTGTGGTTCAG
CCCCATGTCGGAATGGCCTCTGGACATCCGTGGACGTCTTTGCTCGAATCCCTTCTCTCC

CGAGTAATCATTGAGTCATCGATCGATGCCTACCTCATCGGTCGAGGCTTAATAGGCTGC
GATGCATGGTTGTCTCCTGACAAAACTGGGACCCGTCGTCTTGCGTACGATTACGTGGTC
GGCGGAGACAACGTGTTAGTGGCTACTTACTCGTGGACTGGGCTGACCGTGGAGCTGTTA
CAGTCCCAGATTAAGAAGATCTGGGGTTTGGACTGCGGTGGTAAGATCACTGCGGCGGC
ATCAATCTTTACGATTCCGGGGACCGATGGGGTGCTTGGTGCAGAATTTCTGTCCAAGTAC
TTTACTCTCGATATGGTCTATCGCCCGTACGGAGTGTCCCGCTGTATTTCCCAGTGGCCTG
AAGTTCGAGTGTCCTCTCCGGAGGCCTCGTTCGTAAGGACAGTCGCGTTGTTGTATGACA
ACCCGGCTGATCTGGGATTTTACGAGTGGGGTGTGAAGTACTTGGCGTGGCTCCAACGG
GAGTATTTACTATTGGAGGGTAGCCTTGTCTCCATTAATTGGCGTGAGGTCGGGATGATAGA
TCCAGATTCTGCAGGCCAGGAGATCTTAGTACCCATTAGGACCCCCACCCGCCATGACA
TTGA

>Putative Endornaviridae fragment 1

TGGTGTGAAGAAAACGGTGCAAGAAGTGAGTACATACAAGGTGTCATTTGGTTCAATAATC
CGCTCTATAGTTGCCGCACTGCAAGGTTTCAACAACTTGCAGCACATTAATAGCATTGAAG
TGATCAACAATGTTGTCGGGCCAGGTGGTTGTGGAAAATCATCTTACATTAAACAGCACATC
CTTGACAAAGAAAAAACCAGTTTGTTCGCAAAGACCAGATCAGTAGTCAACAGTTATAAACA
TTGCCAATTCAAAGAGTGCGACACTATTGAATCTGCATCGTTGAATAACAAATCACGAGATG
TCATAATAATCGATGAAGTCACAATGGTGAGTCCGTTTGAGTTGATACCCCTTATCAACAAT
CCCGGAGTGAAATTGCACCTGTTCGGTGACACTGAC

>Putative Endornaviridae fragment 2

GGTACGATACAATGCCGAGTTCCATATTGAACTTCCTATCTAAAACCAGTTGGAAGTACCC
AAGTGGTAACATGCCAAGATTGAACAATGGCATGTTGGAGTTAATGTTTGGACCCTACATCA
CCTGCAATAATTGCAGAGCAGTTTCGTGGTTGTGCATCGGAGATTTCATTGATCCCGCAAG
TACAGAAAGTGATTTACTTGACATGCTTGATTGTATGAACGCCGGAGTTTGCATACCCTCAA
ATTGTGATGCGGTGCAGATGGTGACAGCGTGCCTTGGCAACGCTAGATTCAACTGTTACAA
TTGCAATGAAAATGTCTCCACGTTCACTCAAAAAGTGATGCGTAGTGTTACAGTTGTATCAG
AAATTGTGCCTATGGAATTCAGTGTAGGAGTGAGTTTGTTGGAACAGTTCAATGAATGTTTGT
ACATGCAGGGAGCACCACCACGTGATGACATGTTTGCCACGGCGCAAGCCGCATTCGA
CCAACAAGAAAACATCAAAA

>Putative Endornaviridae fragement 3

AGTTTATTGGGTGCGTACAGCAAAACTGCGATGAGGAAACGCAAACCAATTGTTAAAGTGG
ATGCAAAGTCCAAGGCATTGTATTGTCTGCCTGACAACAAAACTGATTTCACTCATGGTTAC
ATGTACGCACTATTAACCAATGTGGGCATCATTATGGCTGTGGCTCTGACGGACTGCATGT

CTGGTCAAACCGTCCTATATCACGGGTTAGCTGTGGACGTGAAGTGCACACTGGCATTGA
AGTTGACAAAAATTAAAACGATGAGCTTGCCAAGAAAATGGCAAGTGATTGACTACAGCAA
ATGTATAACAACCATCAACAATTCAACATTAGAGTACGCCGCATCAATTGGCATACTAGTGA
ACTCAGTTTGTGAACTTGATGCTGATGAGCTGATTATTTACGACACATGGAACAAGAAAAGC
CAAGAC

>Putative Endornaviridae fragement 4

ACTTCTGTATGTTGAGGCAACCATTGGCTATCGAAGGAATATCCACAGCGAAAGTGCATGC
AAATCAAGGCAGTGAAGCACCTAGGTGCGCAGTTTTGTATTGGAATAATAGACCCAACCAA
AACACCATAATGACCAACAGGAAATATTTGTGTACCGCTTTGACCCGTTGCAAGGAATACA
TGTTGTGGATAACAACCACTCAAAACGCTATGTCACCAGTGGCCGCGATTCAAATTTCCCA
CACTGCTAGGGGACTCGAAAACTTGTTTGATTTAGAAGATATGAAAATGAGGAGAGTGGCC
ACGGACCACGAAGTCTTGTTGCTGCCCAAATTGTTAGAAGTTGCAGGAAAGGAGTTCGTTA
GCAAAGTGGAAATGACGCAGCAACAAATCAGCATGACAGTCACGCATCCAATGGC

>Putative Endornaviridae fragement 5

GAGTTGAGCACCCTAGAAATTGCCCGCGAGGGCAAAATCTTTAATATAAAAACAAATGAAG
TCATGCAACGAAACCACGGAACTGTTTTCAAGGTTCGTGGAACGTTGTATCAGCTTGATGA
AGAAACCAAGCTGCATGAAGTGCTTCATTTGTTAAAGGATGCTAAAAAACAACTTGACAACG
GAAAGTGGGTAAAGACTAGTTGGTTGAACAGATCCAATGTGCAATTGTGTAGTTCCAATGGA
GAATTTGGCTTGCCAGCACTTACAAAAAAGAGGTGTCTGTACAAAGAAGACATGTACATTGA
CGCTTTCTTCAATGGAATGGCAGTAGAAGAAGACGGCAAGTGGGTAACTCCAATCAACGA
CATGAACAATGCAAATTGTATTGGAATTCAAACTTTGATCGATGTGTACCATGGAAACCAAC
CCAGCTTGGTCTGTGACAGCCCATTTAGTTATGAGAAATTCAATGGTGACAAGTCAAAATGG
ATCTCAGGCAAGTACTGGATCCCTAAAGCAGATGTACCATCTGCTGCTTCATACAGTTACC
CAAAATACAGGAGAGAGTTGCAAAGAGTGGTCAACACGAGAAAGAGGTACGATACAATGC
CGAGT

>Putative Endornaviridae fragement 6

ACATTTGGCACTAACAGAAGTTTTATGGTGAACGGAAAGACATACTCAAGCGATGTGGAAG
TGTTTGACGAGCAGGTGTTGATAATGCGGTCTGTAAAATACAAATTACTAACAAATTTTATAG
GCGAAATGAGGCACCATGGTGGTTATGGAACATGCGTCACTGGTGCATTTGCATGTTGCAA
ACAAATCATACACCCAGTAGCATTTGACCAAGAAAACAACAAGCGCATGTTTGAAGTACAG
CCACCACTCAAATACAACATGCAAGCAAATATCAACAAATTTAGACAAAACCTGCAAAGAA
CCATTAAGACATTAACGAGCGTTGACATTGGTAACGTGCCCGTCACAGTGCGTTTACCTGA
CAAGTTGCATTTCA

>Putative Endornaviridae fragement 7

CAAAGCGGTGGGTGTGCATGAAGCTGTGATAGATTTCTACATGCTTTGTCATCAGAAATGGT
CATGGAAAGGCACGGGCATACATGGTAACTGGGATGCGATGAGGTTGACTGGTCAAGTTA
CTACGGCACTTGGCAACGCAATAACGAATTTGATAGTACACAACAGGTTTTACAAGAAAAAT
AGCAAGTACATCCAAGCTATGTTCGTACTTGGGGATGACAACATAATATTCTCAGACAGAGT
GTTGGATGTATCTCAACATGGTACTAATACCAAGGACATATACAACATGGTGAGCAAAGTGT
CACAACGACACCACACTGGGGGGTTCTTATCAATGGTAGTACACAATTTGAGTGGCACAC
CAACTTTGTGCCCTCACTTCAAGAGAATGAGGCATAGGTACTCTGTGTGTAATTATACGTTTT
TGCCCATGGATAGATTAAGCAAGGTTGAAGCTAGAACAATGTCATACTGTACTATGCTGGG
GAAAATGCCTCAAACTTGTGACATAGCAAAACAAATAAATCCTGAAGTGGAACTTGAGGAAT
GGTACGATATGGTGAGTGCAGTACAAGCTAATGCGATGTTTGATGAG

>Unsorted Orthomyxoviridae segment 1 PB2

TTCAATATAGACTACGCAACAGTAAGCATGCCATAGTCTGACATACTGTACTTGTGTAACGC
AGCACATTGGCTATCAGGAGCTTATCTATCATAATGATTACACTCAGTAAAGAAATTGATACT
CTTAGTACAGAACATAAACGACTGGTGTCTAGCAATGAATTTTATAACAATAACCTTGCAAAT
GAATTTGATATTTTTCGAAGGTATACTTCAAGCAAGAGCGATTCGAGTCCACAAGGTAGACT
ATGTCAGGTCATACGGAAAAATTGGCCAATAGTATGCCAGAATAACAAGTTTCCTCTGAAAT
ACAATGGGAGAATTTTGCAAAGCACTGGGAGAGAAATTGGAACTAATGACAAACGAAGGG
CCAGCATACATTTTCCTGACTTTTTATCTAAATACGGAGACAAGTGTGAATTGAATGTGGCTA
CCTCATTGATAGAAATGGAATTAGCAAATGTAAAAGAATTTTTCAGTTTAGAAATAGGGGAAA
TGACATTTCGAAACAATAGTATCACAAGAAAACCTGTTCGTACTCAGCCTAGGATAGAAGGT
GTTGCAAGCAGTATGGTAAATGATACTTTATTGAAATTTATTGGAAGTGATGTATTGAGTTCTA
AGACAGAAAGCAATTTGAATGTAGATGCACTGAAAAGGCTGGATACATTGCTGTATGACTTG
CAGAACTATAAATCTTTCGACAGTGTTATTCATATAGCAAGGAACATGTTGACAGAAAAGGTT
AAATGGTTGCCTACTACAGGGATTGTTAATCCTGCTTATTCTGAATTCTCACATATGATCACC
AGTAGATTCCACAGTATCTCAATATCTCAAGAAGTCAGCAACATAGATAGTGGCATTGAGAG
GATCTGCTTTGAAATTATCAGAATTGCATTGGGGCAACATTCTCCTAAGAGTTATTTAATTAAA
ACAATAACTGATATAAAGATCAATGGAGCAGACTTTGAAGTTGTTTTGGAAAGTGTTCGAAAT
GACAAACCTGCCACAAACATCGTACGAGCTGTACTTGGAATGCCTGTCAATATTCAATCTA
AGGTGAGGGATACAACCTTTATAATAATTGAGCAAGAAGCACCTAGAAGACAAGAGATTTC
AATATGGAATTACGGAGCAGTTATGATCCAAAGGAGCTATTACAGATTTCATGGACTGGCAA
AAGTATATTTTGAAAGAAATTCTGTGAAAGGATATTTTATAGTGAGAGACAAGGAGATAATCA
GAATCTTTTTCAATGAGATAGAAAACTATGACAGTTTAGAGCTGTTATTAGACATTGTATACTA
TTGCGCTGCTATAAGAAATGGATTTACTGAACCTTATGACCTGTATATAGACCAAAAAGACAT
TGAGAGGCATTATTTCCAATATTATAAAAGAAGTTACACTAACACTTTAAAGTTTGTAATGACT
TTGAAGTCTGATCATGAGAATGGCAAAATGGTATCCAACAGCTGTATAGTGACAAATAAAAC

ATCAGGATATTTAGAAAAGATTTACCCGTTAACTGAGCCTATACAACCATATGATCAGACTAA
GAGGGAAGTGTCGGTAACAGAAGCTCTAAAAGTATTTGTTGAGAAAGAGCACGGGAAGGA
ATTAGTATTAACCCCTAATCTGCTTAAAAGGCCACAAAGTGCCCTACCCTCTGGTATAACCA
CAGCGAACCCCATTCTAGCAAATCTCTTGGTAAGCGAAATAAACAAAATGAGCTTAATACTC
ACCAGTGTTTTGTCAAACAAGCAAGCCTTTGATGAGTATTTGGATTCTGCAGTTAATGAAAAA
GATAGTGATTACAGAACTGCAGTATCAAGTTCATTCCATCCAACAGAAGCACAAAAGTCTG
CAAAAAGAACCTTAAGTGTTATGTTGAATAATGAAGAAGATGTTTTTGGAAATCCAGCAAAGA
GATACAAGGCAGTCTACTTATCTACGGTGGCAATGACTGCAATACCAGGAACAAATAGGA
CTGGTCTAGAACCGTATCCATCCAATATAACTCTGATTAAGAATAATGAAACTCTTGAACTCA
GCAGGAAGAGAGGATTAGTTACAGGCAAGGGAGAGGAAATTGTGGTCATGGGTAAAAGG
CTTAAAATGCAAATTTCAAACCATGACTACGTATTAATTGGTATTTTCTCAAGAGACAAAGAA
GTTGTCATTTGCAAAAAATGTAGAAGAAGCAATTGAAAAAGGTTATGAGAGGTTTCAGATACAT
ATCATGGGATCATATTATTTTTATCAGAAGAAAGAAGTGACATGGAATATAAGCGACAACATA
AATAACACTTTTAGTGAGTGCCTTAGGGTTGGACATTATTTAGAAATGATTGGCAATAGTTCA
AGTTCGAAATAAATTTTATGTTAAAAGCTCCTTGTTACTTCTTTCTTCTGATAAAAATAATATGAT
CCCATGATATGTATCTGAAACCTCTC

>Unsorted Orthomyxoviridae segment 2 PB2

ATGATCGAGAGAGAACTCGAGCAACCATCCATGCAGTTGATTATTGGGCTACCTTTGGAGA
GTATAAACAGGCAACTATTGAAATTGAAATACAATGGAACAAATAGAAAGACTTATTAAGAGA
CATAGAGAGCTAAAAGCCAGCAACCCAATGTACAGTGAAAAATTGATTAATGAGTATGACG
TATTCAAGAGATATACAAGCAGCAAGAAAGACCATGGACCACAATCAAGGCTTGTTTATAAT
ATGAGAAAAATGTGGCCTATCACAATTGTAAATAAACAAATGTTACCACGCCGGATTGGGAA
GTGGCCACTAGTGGAAGGTGGAGTAGAGATTGATGATCGAGAGAGAACTAGAGCAACCAT
CCATGCAGTTGATTATTGGGCTACCTTTGGAGAAAAATGCAGTGAACAAGTTGCTGATTCAT
TAATTAGTATGCAGATGAATATGGTTAAAGAATTTTTTTCTTTGGATTTAGACAACATTGAGTTC
TGTCATAATTATACGATTAGAAAGCCAGTTACTGTTCAAGCTCCAATTGAGAATGTGCGGGT
GTCTAATGTGAACAACACTCTTATGGGACTTTTTGCACCTGAAGTGTTAGGAAGTAGGAGAT
TGGAACAGATTGACAAAGAAGTGTTGGAAAAGTTGAAGAGGAATCTGTCACCTATAATGAG
CCTCAGATCTACACCTAGCGTAGTTCACTTAGCTAGAAGCTGGATCACCCCAAAAACCAA
GTGGCTTCCAATCTCGTTATCAACAGACAGTAGCACTTCTGAGATTTCTCATGTTATCAGCA
GCAGATATCATCAAATAAATGTTTCTATATATAACAGCAGTGAAGACAATTCAATAGAAAGAC
TATGCGAAGAATTAGTGATAAGAGCAAAAGAGAGTTCGAATCCCAAAGGTAAATTGCAACA
ATACTTAGGAGGAAGCACTATCAGTGGCGTTAGTTTGTCAATAATACTATTGGACTATAGTAA
TGAACGAGCATACACGAACATATGTAGATGTTTACTTGGTTACTCTGTTACAACAGCATCCC
ACGTAAGAGAAACAAAGTTTACAATAATTGATTCAGAGAGTCCAAGAGTGACTTCTAGCAAA
CAATGGAAGTTTGGGATGCATGTGGACATGAGCCATTTTAGTCACTTCAAAGGGTTTGCAAA
AGTCTATTTTGAGAGATTAGGAATTAATGGGTATCTAGAAGTGAAGAACAGGGAAATAAAAC
AAGTATATATGCAGGCTAATAAAGGCGAATCAGTGAAAGAAGTGTTATATGACATTTTATATTA

TTGTGCGTCCATAGAACCAGGATTTGAGAAGCCATTTAGTTTGTATACCAAACCTGCAGAGA
GAATGAACAGTTTCTTCAAAGAACATTCTGCAAATCCAATTGAGTTTTATAGAGCTTATGGGG
TTGACAAACAAGGGGTAGTTAGTAACGACCTGCACTACAATGTGGAAAGCAGAGGCTATTT
GCAAAAAGTATCCAAAATATCTCAGCCAATACAACCGTATGATCAAACCAAGTATGAGACTT
ATTGTGATGACGCCTTAAATGTAGTAAGAGTTGGCGCTCAAGGTAGAGAAGTTGTTATAAATT
CCAGCAATATTAGAAAAACTGATTGGCCATTGCCAGTAGGTCTGAAAGTTGTTCATCCGGA
GCTAAACAATATAATCATAAACCCATTGAAGAAATTCCAACTTGTCGCAGAGTTTTGTCTAGA
AAATGGAGATCAAATGGAGAGGAGCATCATGAGTACAATAGGAGAAGAAGATGATCAAATA
AATAGCACTATATCTAAAATACTGGAACCTAGTAGTGGACAACGAGCAGCTAAGAGAAAGT
TAGAAGCAATCATTGACAATGATGAAGATTGGAATTTGAACCCAGTAAAGAGATATAAAGCA
TTCTACCTGAACTTAGTGGCTAATGCTCCAATAGAAAGAGAAAGTCATTCTACAACTGGAGT
GCCGCTTTCACTTAAACTCCGTGGAACAAGAGACGTAATACAGCAAAGTAGAAAGCGGGG
TCTGGTAACTGGTGGGCAAACTCAAATATTGATAATGAACAAGAAGATTAGACTTGAAAATG
ATTACACATTGGGGTATGCTCAAGTAGGTGTTTATACACAGAAGCCTATTATAGAGTCAGTA
GACAGTGTAGAAGAAGCTAAAGAAAAGGATTTAAATAGATTCTGCATCCAGTCAAAAGGAA
GATATTTTGTTTTTGAAAAGAAAGAATATATATGGAGTGAGAGCGACAATATAAATAAAGTGGA
CAGAGATCAATTGAAAGAGGAAAGAGCTCAAGACTTGGAAAGATCATTTGCTGATATTCTTG
GAATTTAACCATTTCATTCTGAAAGATGTTATTATTAGAAAAATTGCCGTGTTACTACTTTGC

>Unsorted Orthomyxoviridae segment 3 PA

CCTGACGTTCGGTAGTTATAGACGCTACTTGAGGTGAGTATTAACAGGAGCTTAAAACAATT
TTATCATAACATGCGTAGCAACATACTCGGTTTTAACACGGAAGTATGGAACCTTTCAGAAA
ATTACATGGATTGGAACTCCCTGACGTTCGGTAGTTATAGACGCTACTTGAGGTGTATGTTA
TTTGAAGCTTGCTCTCAAATAGCAAATGATCATTATTACGATCACCAAGCGCGTTATTCAGTC
ACCGTTTTTTATAATGAAGAAAAAATTTATGGACAGCTTGGATTGTTGAACATGCCCAAAATT
GAGAAATTTACACCTCATATAGTGGATAAGAAATTGCGGATTCAAGGGAGGATGATTTTTGTA
TCTGAATCTGAGTTTGAACAACAAGTTGCCATATACAAGAATGATATTATAATGCCCTCAGGA
TTTTTGATTGTGTCGCTCTCTGGTGAAGTTTACAACAAGCAATTTCCAATAGATGACAGTCAA
AAGACATCTATCTCACAATATGTATCTTCGAAAGTTTCTAGACTGAAAGAACTTGGATTATTG
AAAGAGTTTGAAGAGGCAGTAAAGACTAAATTTGAGAAGAGAATTTACCTTGGTTCTCGCTT
CAAGAATTTGCTCACAAAATCTACAGAAATAGGTATATTGGAATCTTCCAGACCAAAAATAA
CCGAGATACCAAAACTGGAGGCCAGGCCTTGGGTCGAGCTAAAAGAGCTAAAGAATTTCT
CCCTCAATGAAGGAAATAAAGAAGCTGAATGGAAAGCTACTCTAGTTGGGTCTGAATCGTC
TCCTTTATTCTTTGACAGTCCAACTGACCAAGGTTTATGTTATAGCTTCATAAGCTCATTTCAG
TCCTTTGAAATCATTAGGAGACAAGAGAATTACTCAGGACGGGAAGAGACGAATTCAATAAT
AGAAAGCTTGAAAGAATTTGCTCTAAATGAGAACAATAGAAGACAATACAAAAGTCATGAAA
GGCAATGCTTTGGAATTGGAAAGAAAACCAGAATAAAAAACAGAGAAGAAAAAGAAATTGC
TAGTGTAACAGAAAGATTCAAATTTGATCCAGAACTCAGAACATCAGCAAGATGGGTTGATG
AAGAATTGGAGTTCCTTAGCAAAGAAACAAGCTTTAAATGGACATATTCAGAGAGAAATGCT

ACTTATAACAAATATGATGAAACTGGAGAACAGCTAGTTGACTTATATCATGAAATTATCGGC
AGCACTATGGCTTGCGCTATGATAGAGAAGTGGAAGACCGCGTTTTCTAAGATTAATAATTT
AATAGGCCAAGATAGAGAAAAACTCACTTTTGTGCCAATAACCAACAGACGTAATATTGTAG
GAGAAGAAAGAACAGTTCTCTGGGGATTTGTTATAATGGGACCTCATCACTTAAAAAGAGAT
ACCGATAAAATACCACTTCTCATAATGGAATTCGTTAAAGAAGACAACCCAGACAAATATCC
AAAGCATTGCTATGGCAAGATGCATATGAACTCCACAGGAGAGAAGAAATTGAACCTTACT
TACAACTATATGATCAAAGTTACTAGTATCAGCAAATACAAGGCACACATATTCTCAAATATA
GGACGAGTGGTTATTCAGCCTTGTAGTCTTTTCTCCAAAGTGTTACTAAACAACGCAGCTAA
CTCAGGTATGCTTAACTTGGAATACCGGCCTTCGATTAAATTAACTATGAATGACAACTTGGT
GGAGCTTGATTTACACCATTGGATTAGGATAGTATTATCTTTGGAATTTTTAATGGCGATTCAC
AATGATTCACAGATGGAGGGAGGTATTGCGAATTTGAGAAGGCTGCATATGATACGTCAGG
CGTTGTTAGAGAACAGGCGAGTCTTTCTACCAGAAGGAGGGGACCCATTCCAAAAAGTTC
AAGAATGTTCCACTTCAAACCCAATTTTACTTTACCTTATTTCAGGCTGGAATATGATGTCGA
CTAAATACTTGAGAGAAGATTAAGTTAAAAGCTCCTTGTTACTACTTTTATTAATCATTACCTC
ATGATCAATTCAAACCAATGAAATATACTGATCGAGG

>Unsorted Orthomyxoviridae segment 4 PA

AAAGAATACACGAATGCTTAATTTATGCTGTGTGCTGTCAGTATAAACAGGCAGTTATTTAAG
AGAAATACCATGAATAGACCAGAGTATATTGATTCAATTATTTGGGAAAGGTCAAGACTTACA
CAGGAGTGGAACATAATAACTCAAAGCCGTTTTAAAAGAATACACGAATGCTTAATTTATGCT
GTGTGCTGTCAGATTTTTGATGAAGAAGTTTTTAATGACAAGCCTAGATATTTTGTGCTTCACA
TGCTTAACCCTAGTCACAGAAATGCTTGGTTGAATAAAAATGGATTCAGCTTTGAAGAAGAA
CAAGATCTACATTTATATGATTACAGGGAACAACAGATGTATGTGGTGAACATAAAGAGCCA
ATTTGAGGGAGATGAAGTTAAACCTTATAACAATGGAAGAGTGGAAACTCTTGATTTTTATTAT
GATGGAACTATTTCCGAGAGAACAAAGACTTTGTTGTCAAAAACTCAGATGCAAGGAGTAG
CTGATAGTATAATAAAGGTTTCTAACGGCTTAATGGTGGAAGGTGAATTTTCTAATTACATTGA
GGCTTTAACTGCTGGATTTAAAAAGAAATTAACTCTGAAGTCAAGATTTAAAAGTCTCCTAGA
AGAGAAAACGGATTATCCCTTATGTAGCACTGAGGACTACTTAGATAAAAAGCCAGCGGAT
ATACATGGACTACAGTGGAATTATATTAAAACTCTTCCAGTGTTTGACTTGAATGAAGGGTGC
CCAGAATCACAATGGAAGCATTTCTTAGTAGGCACAGAATCTATTCCATATATAAGCAGTTAT
GATTCAGACCATATTATAATCACTGAGTACTTTAGCCAGGTTGAAAGATTTGAGATAATAAGG
AAGACAAAACATAGTAGTGGAGAAGAAGAGGTTGAATCTATACTTTCTAGTGTGCATAAGTT
CATGGAGAGCTTGAATACAAGAAGATGCAAAGTGAAAACTGATGTGAAATGTTTTGGAATTG
GAATTAAGAATAGAGAGAGAGCAAAGAAAAATTTGACTATCTTAGAAGATTGGGTTAGAGCT
GATTTCCCAGTTGAGAAGAGGAGCTTTCCAACCTGGATGAGAGAAGAGGTGTCTACTTTGG
AGTTGCATACAAACACAAAGTGGTTAAGTTTGGAAGAAAACCCTACATATAATGAATATGATA
GGACTGGTGAAAAAATGTGCACAAATTATTTGAAACTTATTGAGAATAGTTTTTGTGCTGCAAT
GGTGGAAAAGACTCAGATTGCGTCGAGTAGAATGTATCAAGAGGTTCATACTGATAGGGCT
AAAGTAACCATGGTGCCAATAATAACACGGAAAGTAAATAGTAAGAATGTTTCAGAATCCCA

GTTTTGGGGAATAATAATTGTCGGAGAGCACCATGTGAAACAGGAGTCTGATAGAATACCA
ATAGTAACCTTAGAGTTCGTGAGCAAAGATAACCCTGTCAAATATCCAAAACATTGTTACGG
CCAATTTAGAGTAAGAGATAAAAACCTTTCAGATAACCACAATGTTTTGAATTTCTTAGTGAAA
GTTAGTTCTATCTCTAAAGTAAGGTTGCACATCATGAGCAATTTGAGAAGAGTTTGTTTGCAA
CCTGGTAGCATATACTCTAAGATGGTGCTGGAGAAAAGTGCGAGTGATGGTGTGTACAGTG
AAGGATACAACCCCTCTGTTACATTATTTATGGATGGTGGCAAAAGAGAAATAGATATGAGC
CTCTGGGTATTGAAAATATATTGTCATGAATATTTGATGGCAGTCCATAATGACTCTCAAATG
GAAGGATTTCTAGCTAATATGCGGAGATTACACATGATAAGACAAGCCTTAATGGAAAACC
GAACTGTAAGTGTGCCCCATGGAGGAAATCCAGGATCTAAGGTACAGGAATGCATTTTAAA
CAATCCAATGGTACTATTTCTTGCTAGTGCATGGAATGAGATGCCTAATGTATATGGTTAATG
TAGTTAAAAACTGCACACTCTAATTTGTTCAAGGTAATAAATAGTTGGGCATGCGATATGGCA
CACCCGATTAAAGGCACACCATACACATT

>Unsorted Orthomyxoviridae segment 5 Nucleoprotein

AACTGTGAGTTGATAGGGTCACTAGTAATGGCCATGTTTAAGAATTATCAGAGGCTTATTAAA
ATAAATTAGCAACTGTTAAGCAAAGTAACTTTGAAAAACTCGAGATAAAATGGAAGGCATTG
AAACTGTGAAGTACGATGTTGATCCTACTGCTACGGCAATGAAATATGTTGCCTTTAAAAAGT
TCATGACCCTGATTGCTGGTAATTTGGACATGGTAATTGATTCAAACTGTGAGTTGATAGGGT
CACTAGTAATGGCTATGTTTACGGATAATACTAGTTTGAAGATGGAGGATGAATATGATGTGG
CTTACTTTGATGAAAGTAACACTGTCAAGTATAGAAAAGCTAAACTAAGCATTTCAGCTGCTA
AGGCAGAAATCGACAAGATAATGAGAGATAATGAAGAGCGTAAACCTTGGATGCCTTTTCT
GGCAGCGTTGCAGTTGGCAGTAAAAGTCAAAGATTCCATTCTTTGGCAAGACAACAGAACA
ACTAAGGAGTTAATGGTTTCGCCCGTTTGTGAGGTTTTCGCCAACGGATACAACGTAAAGA
ACAAATTGAAGAAGTCTAGACAACTTAGTTTTGGTCCATTGGTACACTTGGATCAGTTAAGG
AAATTAAATGCAGAATCGAAATCTAGAAAGCTGAGAGATTCAGCAAGAGACAACATTAAAG
CAAGATTGCTAAAAGTGCTTAATAGACAAACAGTTGGCAGTGTGCAAAGAAGTATTTTGAGA
GAAATTGCTGAAGGGAAGTTTCAAAATCTCAATAGTCTTTGTTTGAGCTATCTATCAATAAAG
CCCCATATAGAACACCATTTTGTCCTTACTTATCCATTTATTGCTAACACTGTCAACTTTGAA
GGAGCAAACTTTACTGATGAATGGGTATGGAAGAAGGTTTCAGAAGCTAAAATTGGTATTAC
AGCAAAGAATCCAGAATGGGCTGAATTCAAGTCACAGATATTGATACACAGTGTTTTCCAAA
CTGCTGGTGAAGATTTGGGAGCGCTCAGCCATATATTCAACAGGAAATTCTTCCAGAGGAA
AGCATTCGGAAAAGCCCCTCCAAAGAGAGAAGAAGATCAAATCTTTATTTTCAAGATGCAG
TACTCATGTTGGACAAAGCCTCAAAGAGGAGCTCCAAGATCAGTAGAGTACAAGTCCAGA
GGTCAAGTTTGTTCTAGACCATCCCTGAGAGGAGCTAGGGCATCTCTAAACTCTTTTGGAA
GTATAGCAGAAATGAATCATGCAGTGTCAGATGGAACAAGCTCTGAGACATTGTTAGAAGC
TCTTAATAAGGAATATGAAGAATATAAGAAGATAGTTCAAGAAGGTACTGGAATCTTTTATATT
CCGGGTAGTGAGAATAAGAGTCCTTTGAACATTATGAATACTGGAGAATACTTACTTGGTAA
TTGAATATTTTCAGTAGACATTATTATTGGTTTTGGGAATATTTTGATTAATATATTATTCTATAGC

GTAACAGAAAAGTATTGAAAAAGCCTCTTAATAAGGAATATGAAGAATATAAGAAGATAGTTC
AAGAAGGTACTGGAATCTTTTATATTCCGGGTAGTGAG

>Unsorted Orthomyxoviridae segment 6 Nucleoprotein

AATCAGTCTAAGTCTTAGTAGAAACTCTGAGATTAGTATAAACAGGCAGAATAGAAAATAAAT
TAAACCTTATTCAAATAGTTAATTTTGTGAAAAATGGCCGGAAAGATAGTCTTGGATACAAAAT
TGACAGAGAAATATTATCAAAAGTTCAAAAGTTATATGACAACATTAGCTGGGGACACAATC
AGTCTAAGTCTTAGTAGAAACTCTGAGATTATAGGCAGTGTCGTAATGGCTCTTCTAACTGG
TAACGGAAATGTTAAACCAGAGCAAGAATACAAATTCGTACGTGTTTTTGCAAGTGAGAACG
CACTAAGATTGGAAGAATTTCAGTCTAAATTGTTAATCAGTGCAGTGGAACCATCCATAAGG
ACAATGATGACGGACTCTGAAAACAGGAAAACATGGATGCCTTTCTTGGCTGCCTTACAGA
TATCAGTAAAAACCAAGGACGACATTATCTATCATAAAGCAGAAGTCTCTAGAGATTTAGGA
GTACCAAGTGTTTGTGAGCCATTTGCAACCGGACATAAAATAAAAGACAAACTTAAGAAGA
GTAGACAGATGAGTATTGGGCCCTTAGTGCACATACAACAAATAAAACGGTTAGATAAAGC
AACAGGAGTGAACAGGTCTAGATTAAATCCTACTAGTAGAGCAGCAATTAGAGAAAGATTGT
TTGGTGTTCTCAAGAGACAATCTATTGGATTGGTACAGAGGAAACTGATAGATGAATTCAAA
GCTGGTAATGAATCAGTATTGAGTACATTGTGTCTTAGTTACTGTTGTATAAAACCTCATATTG
AACACAATTTTGTTTTGACGTACCCCTATATTGGTGTTATTGACAATTTTTCTGGTGCTAATTTT
ACTGATGAATGGGTTTGGAAGATTGTAAAACAAGCTACCCCAATGATTGGATGGATTGTGTA
TGGTAGAGAATGGTTGGAGTTCAACTTTCAAACTGAGTTGCATTGTATCTTCCAGACCAGTG
GAGAGGATTTGGGAGTGCTTTCTCAAGTCTTTAATAGACCAATGCTTCAAAGGAAAAGTCTG
GGAAGACTTAACAAAGAGGCGGCAATCTATGGTGACTTCATAAAAGACTTCAGTTATTCATT
ATGGTCAAAGCCCCAAAAGGGAGCCCCTAGAAACTTGAATGGTGGAGTCCGTGGTCAAAT
TTGCTCAAGACCATCTTTGAGAGGTAGCAGAAAGACTTTTAACAACTTTGACAGTCTTGAAC
AGCTTGAGTCTTCCTATATGGTAGAAGGTGCTCAGAACTATGTGGAAGCTATACATAAGGA
GTTCATGGAATACAAGAAGCTTGAATACGAAGGTACTGGTAAGTTTTACTTAAG

>Unsorted Orthomyxoviridae segment 7 Glycoprotein

TAACAACAGTTCAGCAACTTGTAGAACAAGTCAATATGAACAGACTTGAGTCAGAATACAAT
GCTATAGTGAATGAGAATAGAATCAGACGTATAACTGAGGTCATGATCGAGATGATCGCAG
CGGTTGTGGATATAGACAAACATCTTTTGGGAAGAGTGATAAACCAGCCGGTGGTAGCACA
TAGAGTAGGATTAGGTTTGTATGAATTGAGAAGACCTGATCTGGAAAATTTAGGAAACACTCT
TTCACTTCATACTTATACTACAAATGGCCACTCTGAATCTTTTGGTATAGGAAATACGCAGTTT
ACTCCAATAGATATATGGAATTCTACCTCACTAGTGTCGTTCAACGTATCCAATTTAAAAATT
GAATCTGTTGTAAATGACACAGTGAATGCAAATTACCTGGCGTCTATACATATGAGAGAAAT
GATGGAATTCCTTAGAGGAGGTAGTGTAAAAAGTAGCAGTTTAAAGGATGTGTTAGAAATGC
CTAGTGGTTATCTGCACACTTTGCTCCATTTGATATATGGGTTAACTGCTGCATTAATGGTGA
TGTCTGTAATTGCTATCTACTGTGGAATAAGATTTAGAAAATCCATACTGGGAGGATGATTTT

GGTTCGCCCTCTCGTTTACTTTAATAAATTGTCGTTCGATTAGAATAGTGATATCAATGTGTAA
GAAATTTATGTAAAAACTCCCTTGTTACTATTATCAGGGAGTTATTTGAATAATATTCTTTATGT
GAAATTGAGTATCCGCCATCGACATGTACTTTAAATTACTTTGTTATACTGTCTTGCCCTTTAT
TGGATGCTCTAGGGTGGTACAAATTAACCCACTAATTAAAAGTCTTATAGACGACGTAAAAA
CGAGAATAATTAAACATGAAGTACTGGAATTGCAACAAACTCATGTAACAATGTTTCAACTAG
ATAGGAGCCTATTGAAACATTATTGCTTCAGTGGAGGCCCTTGGGACAGCAATACTGGTTG
TGAAAGTAGTTTGGTCAACCGTCCTCCGGGATATAGCGAAGCGGTAGTATGGGATGAAAC
AAAGTCTTGTTTCACAGGAAAAGATTACATTGATTCTTACGGAGAAAGTTCAGACATTTGCTT
TAAAGAACAAGAAATGGACATACAACACCATGTAGCCAAGGAACTTCATTTGAATATTTACA
ACAACCATTTTTGGCGTCATACTTGCACATTATCCTGGTCATGTTCAATAAAACGGGTAGAA
GTACCAGTATTTGTCAGCTACTCTAGAAACAGTGGACACCATTCCGTGTATATGGATTTGTT
GAATGGCACTAGAATCTGGTTGAGAGAAGGACTCGGTACTCTTTTCTTTAGCGGAGCCAGC
TCTTTCTTAATTTCTCAGACACCTCCACTGAAGAAGAGAAAGGCCATCTCTAGATGCTTGTT
GAAGGGGAAAGACACTGAATGCCAACTTGATGATGCGAGCGGTACTATCTACAGATGGGA
ATCAAGAAATAATTGTTTTGGAAGAACATGTGTCTTAAATGAAGGGTTTGAAGAATCAGATTCT
CACACAGCTGCAGAAATAACAACAGTTCAGCAACTTGTAGAACAAGTCAATATGAACAGAC
TTGAGTCAGAATACAATGCTATAGTGAATGAG

>Unsorted Orthomyxoviridae segment 8 Matrix

ATGCAGGCATTACACAAAATTTTAACTGAATCTAAAAAACCATTCAACGAAGTGGATCTATAT
GTTGGCTGGGGAAAAGAGATAATAAGAATCATGTCAAAGTCAAACATTTTCAGTATCAAAGG
AATACAATATGAACTTCTCAAAAACAGCTCAGATAGAATTACATCACTGTTAAATTCTTTTTTG
GAATAGTTGAAGAGGAAGTTTGTTCCACTCTAAAGGCTTTGGGTTGGAAAAAATGAGAAAAT
ACTCTTACAATGTTTGATCTCTTTATTTCAATTCTGTAAACTTTATAGAAATGGAGACAGTGAAT
ATTTTCGAAGTAATCCCTAGAGAATGTCGTTCTGAGGTAAAGATGATTTGGGAAGCCTCTAG
TTATGCAGAAAAAGTCTCCATGTCTAAGTTATTAATGCAAAAATTTAGCGATAGCTCAAGACT
AGTAGTCCTATTGCACATAATAATCTTGCATCAAACTGGAATGAAGGCTGAACTTGGGAGTA
AGAGGAAATGGATGTTGACATTGGACGTGAAAAAACTTAAAACAGTCATAGGGAAAATGAA
CATTGATGAGTTCATAGAGTTAATGTCTAGATTTAACAGAGAAGATTTCTTTGGGTTTGTTATG
TTGGAATTTAGTAAATACAAGGATATATATAGAAATAAGAATGCTATTACAAGGATGATTCATA
TCCTATATGGACAATCTGATTTAGCATTGGATAATGTAGCTTGGATTGAAGCTGTTGATGCCA
AGAAAGATTACAAGAAGGAGAGTGAGAAATTTGTCATAGATGGGCTGATAAATGAGATGAA
CCTGTTGACACAAATGTCCAAAGCAAACTTGAGCACACCGGAACTCAAACAATGCCACAA
AGAGATTAAGGAGACGACCATGGAGATGCAGGCATTACACAAAATTTTAACTGAATCTAAAA
AACCATTCAACGAAGTGGATCTATATGTTGGCTGGGGAAAGGAGATAATAAGAATCATGTCA
AAGTCAAACATTTTCAGTATCAAAGGAATACAATATGAACTTCTCAAGAATAGCTCAGATAGA
ATTACATCACTGTTAAATTCTTTTTTGGAGTAGTTAAAGAAGAAGTTTGTTCCACTCTAAAGGTT
TTAGGTTAGAAAAAATGAGAAAATACTCTTACAATGTTTGATCTCTTTATTTCAATTCTGAATTTA
ATGAAAAACTGCCTTGTTACTACTCCAAAAAAGAATTTAACAGTGATGTAATTC
